# Photo‐Activated Merging Cascades in Droplet Swarms Upon Synergistic Solvation of Surfactants

**DOI:** 10.1002/smll.75101

**Published:** 2026-08-13

**Authors:** Priyanshu Singh, Roël M. Vreuls, Gerco W. A. Meeussen, Peter A. Korevaar

**Affiliations:** ^1^ Institute for Molecules and Materials Radboud University Nijmegen The Netherlands

**Keywords:** active matter, droplet‐based logics, emergent behavior, motile droplets, systems chemistry

## Abstract

Chemically active droplets at fluid interfaces provide versatile building blocks for dynamic matter, yet most systems are limited to behavior encoded within individual droplets or simple pairwise interactions. Here, we report a strategy to generate non‐linear, cascading behavior in droplet swarms by combining photo‐activation with synergistic solvation of surfactants. At an air/water interface coated with a crystalline layer formed by a decylammonium/carbamate ion pair, mesitylene‐based droplets containing a photoacid generator remain immotile until UV irradiation locally destabilizes the layer, which induces droplet motion via capillary effects. While both UV‐responsive mesitylene droplets and tri(ethylene glycol) monododecyl ether (C_12_E_3_) droplets are individually immobilized within the layer, their fusion produces hybrid droplets that disintegrate the layer through synergistic solvation and exhibit enhanced motility that persists after UV irradiation is stopped. This non‐linear activation enables cascades of chasing and merging events that propagate through large droplet fields well beyond the initial space‐time window of photo‐activation. By introducing repellent boundary structures, these cascades can be spatially confined, allowing control over collective swarm dynamics. Together, our results demonstrate how synergistic solvation allows to program emergent, multistep behavior in active droplet swarms, opening avenues toward droplet‐based logics in chemically driven self‐organization.

## Graphical abstract

UV activates the motility of UV‐responsive droplets through a decylammonium/carbamate crystalline layer at the air/water interface. Upon merging with immobilized C_12_E_3_ surfactant droplets in the layer, hybrid droplets form which dissolve the layer due to synergistic solvation. In large droplet fields, this non‐linear behavior drives cascading events of chasing and merging that propagate well beyond the initial space‐time window of photo‐activation.

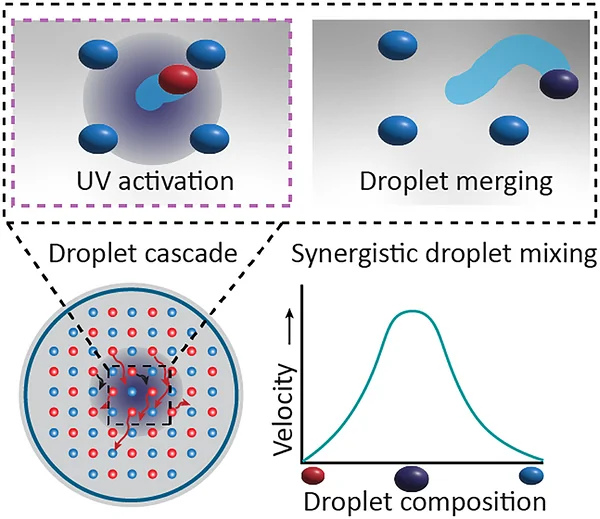

## 1 Introduction

Droplet‐based logics offer versatile design principles for creating dynamic matter, in which chemical reactions and physicochemical interactions direct the motility of droplets and enable their organization as building blocks. While systems relying on individual droplets are constrained by the behavior encoded within a single entity, logic‐based interactions enable swarms of different droplet types to collectively process input stimuli and display emergent behavior richer than the sum of their parts [1, 2, 3]. These interactions can be non‐reciprocal [4], such as when droplet type A chases type B but not *vice versa*, producing predator‐prey behavior [5, 6], or non‐linear, when droplets of types A and B merge to generate a new behavior C that neither type can achieve alone. Such concepts expand the functionalities of droplet‐based systems –featuring applications as diverse as sensing [7, 8], optics [9], computing [10, 11, 12, 13, 14], maze solving [15, 16], cargo delivery [17, 18, 19, 20], multiphase reactors [21, 22], Janus droplets in food industry [23], reconfigurable liquid architectures [24, 25] and biomimetic systems [26]– with autonomous chemical decision‐making [27]. For example, by initiating downstream processes without external control or by opening up novel routes to build up non‐trivial droplet assemblies at the micro‐ or macroscale.

Optimal control over droplet behavior [28, 29, 30], that is, their motility or acquisition of non‐equilibrium shapes, critically relies on physicochemical interactions amongst the droplets through their surrounding bulk phase [31]. To this end, air/water (a/w) interfaces provide an attractive medium, as surface tension gradients allow for controlled Marangoni flows to direct the pathways of floating droplets [32, 33, 34, 35, 36]. Earlier, we reported crystalline surfactant layers formed at a/w interfaces upon deposition of the surfactant decylamine (DA) as a means to facilitate droplet motility and setup non‐reciprocal droplet–droplet interactions [6]. Aldehyde‐based oil droplets react with DA in the layer, causing the droplets to move through the layer while leaving behind an open channel, which is then avoided by other aldehyde droplets, giving rise to a “memory effect”. Moreover, when tri(ethylene glycol) monododecylether (C_12_E_3_) droplets are deposited in the crystalline layer as well, the layer initially suppresses their motion. However, the aldehyde droplets create surface tension gradients upon depletion of surfactants from the a/w interface, thereby attracting the C_12_E_3_ droplets. As a result, the C_12_E_3_ droplets were observed to chase and ultimately catch the aldehyde droplets along the channels they had created in analogy to “predator‐prey” behavior.

Here, we report –departing from these interaction motifs amongst two droplets– a strategy to trigger cascades of multiple chasing and merging events in droplet swarms, driven by non‐linear interactions of different droplet types with a DA‐based crystalline layer. At the core of our approach, we exploit the synergistic solvation of two non‐solvent droplet types that individually do not dissolve the layer, whereas upon mixing, they dissolve the layer and become motile (Figure 1a). As building blocks of our system, we first grow stable, continuous crystalline layers [37, 38, 39, 40] based on DA at an a/w interface. DA that is dispersed in an aqueous solution reacts with atmospheric CO_2_ to form an ammonium/carbamate ion pair that constitutes the crystalline layer [41] (Figure 1b). Second, we establish photo‐controlled motility [42, 43, 44, 45, 46] of mesitylene‐based droplets that remain in position when deposited in the crystalline layer, and only start to move when exposed to UV (Figure 1c)—exploiting the pH‐dependent stability of the decylammonium/carbamate ion pair as the carbamate dissociates under acidic conditions. Third, we explore droplet merging cascades, triggered by the fusion of a UV‐responsive mesitylene‐based droplet with an immotile C_12_E_3_ droplet, resulting in a reactivated droplet with enhanced motility (Figure 1d). Together, these basic concepts allow us to establish large fields of droplets that are kept in position by the crystalline layer after deposition. Next, photo‐activation of a subset of the droplets triggers unique cascades of droplet merging events, driven by the formation of droplets with the right chemical compositions to readily dissolve the crystalline layer and thereby sustain droplet motility beyond the UV activation time and space window.

**FIGURE 1:**
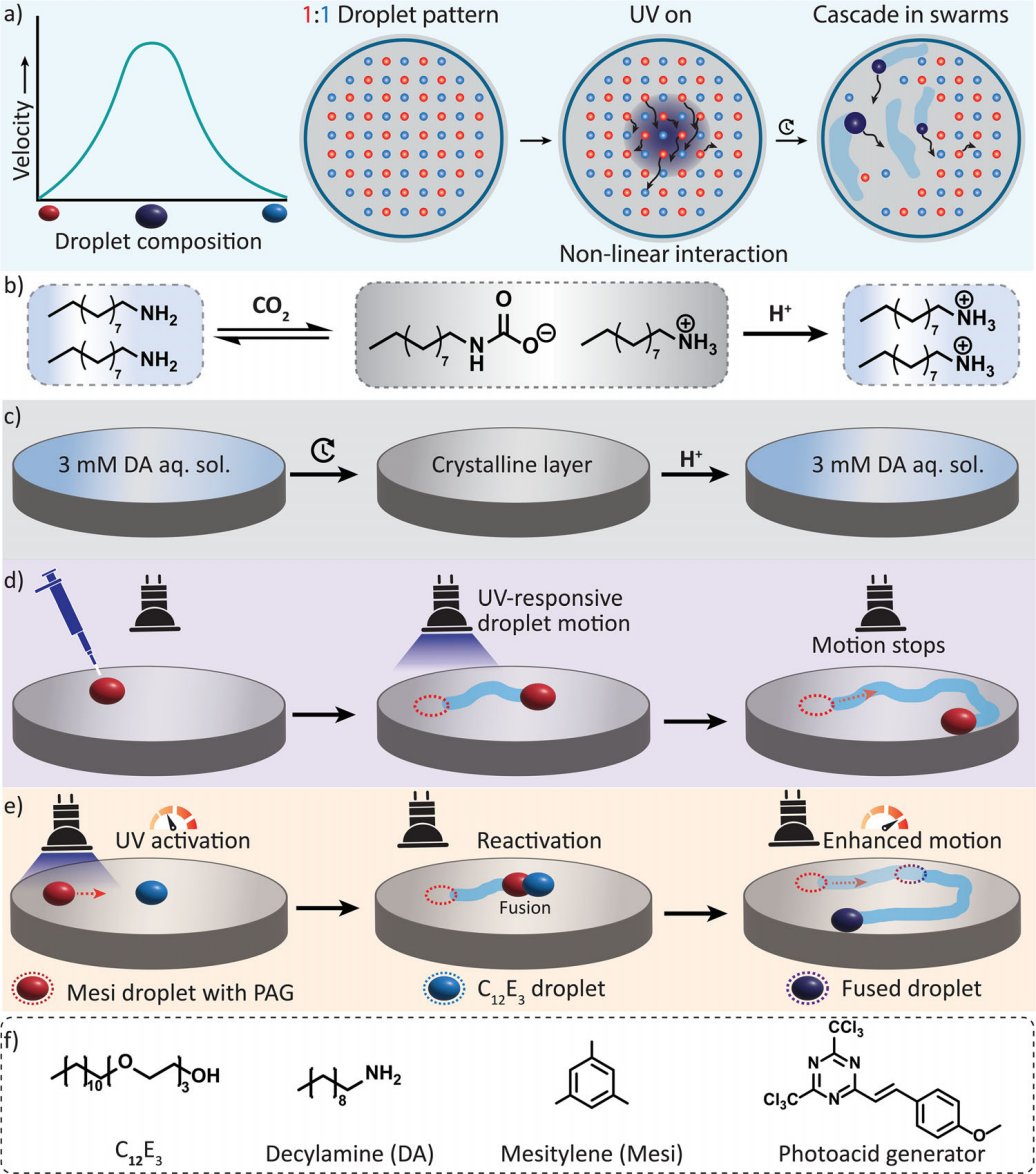
Design of chasing and merging cascades in droplet swarms, exploiting synergistic solvation. (a) Two droplet types that individually do not dissolve a decylammonium/carbamate crystalline layer formed at an air/water (a/w) interface become motile upon mixing. This enables large fields of droplets to remain immobilized after deposition, until UV activation of selected droplets triggers merging cascades. (b) Decylamine (DA) reacts with atmospheric CO_2_ to form the decylammonium/carbamate ion pair. Under acidic conditions, the carbamate dissociates, yielding a decylammonium ion. (c) Formation of a stable, continuous crystalline layer at the a/w interface from an aqueous 3.0 mM DA surfactant solution, which disintegrates under acidic conditions. (d) Motility of UV‐responsive droplets, comprising mesitylene with a photoacid generator (PAG): after deposition they adhere to the crystalline layer and remain immotile until UV irradiation activates their motion, generating millimeter‐wide channels through the layer. (e) Non‐linear, cascading interactions between UV‐responsive and tri(ethylene glycol) monododecylether (C_12_E_3_) droplets: Fusion yields a hybrid droplet with enhanced velocity. f) Molecular structures of the building blocks of the system.

## 2 Results and Discussion

### 2.1 UV‐Responsive Droplet Motility Through a Crystalline Surfactant Layer

As a medium for our motile droplets, we exploit the crystalline layer that is formed at the air/water (a/w) interface of an aqueous decylamine (DA) solution. When a 3.0 mM DA solution is poured in a Petri dish (3.5 cm diameter), a continuous crystalline layer spontaneously forms that covers the entire surface of the solution over a time course of 300 s (Figure 2a,b and Movie S1). Infrared (IR) and ^1^H NMR spectroscopy measurements performed on this layer material after isolation from the solution reveal the presence of a carbamate, which readily forms upon exposure of the DA solution to atmospheric CO_2_ (Figure S1 and S2) [41, 47]. The formation of ammonium/carbamate ion pairs as a mean to stabilize crystals [48], assemblies or gels [49, 50, 51] is well‐documented in literature. Importantly, we note that our DA‐based crystalline layer disintegrates both at low pH (where the carbamate is expected to dissociate back into a decylammonium ion and CO_2_) as well as at high pH (where the decylammonium ion deprotonates), as we reported earlier [6]. As a result, only at intermediate pH where the decylammonium/carbamate ion pair can be expected, the layer is formed. Importantly, the crystalline layer is smoother and more stable in comparison to the layers that rapidly form by applying pure DA microdroplets at the a/w interface, presumably due to the absence of strong Marangoni flows emerging from the DA microdroplet [6]. Furthermore, the presence of DA in the aqueous solution allows the layer to regenerate (*vide infra*). Thereby, crystalline layers that slowly grow from a DA solution upon uptake of atmospheric CO_2_ and formation of the decylammonium/carbamate ion pair provide more continuous media for droplet swarms to propel through.

**FIGURE 2:**
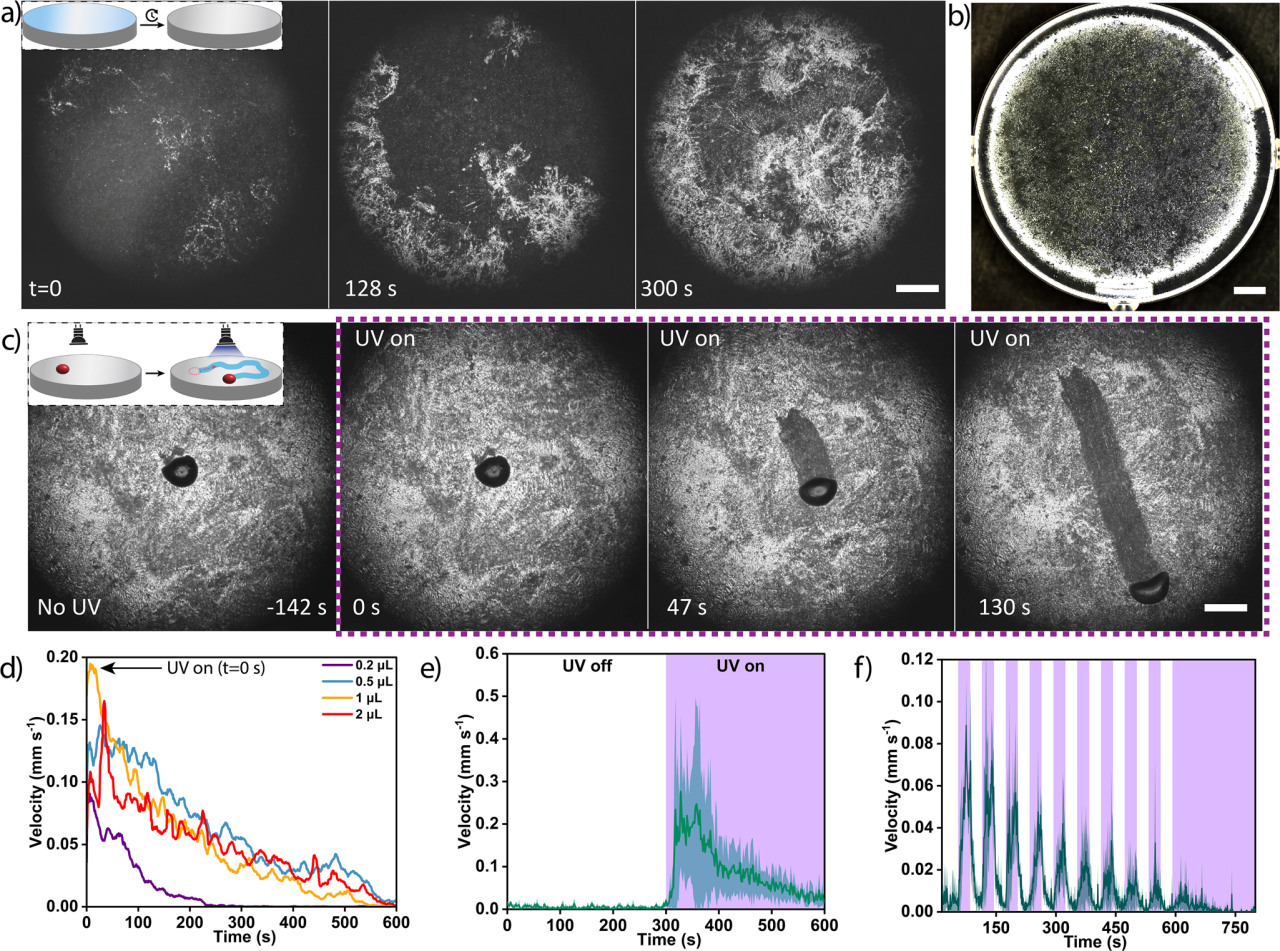
UV‐responsive motility of droplets through the crystalline layer. (a) Optical microscopy recordings of a 3.0 mM DA solution forming a crystalline layer at the a/w interface over time. The scale bar represents 2 mm. (b) Photograph (top view) of the layer, covering the entire surface of the aqueous DA solution in a 10 cm wide Petri dish. The scale bar represents 10 mm. (c) Optical microscopy recording of a 0.5 µL UV‐responsive droplet deposited at the a/w interface with a crystalline layer. The droplet adheres to the layer when UV is off (*t* = ‐142 s) and starts to move when UV is turned on (*t* = 0 s), creating an open channel at the a/w interface. The scale bar represents 2 mm. (d) Velocity versus time profiles acquired on UV‐responsive droplets with different volumes, exposed to UV from *t* = 0 s (*n* = 4 experiments per droplet volume). (e) Velocity versus time profile acquired on UV‐responsive droplets (1.0 µL), exposed to UV from *t* = 300 s (*n* = 4 experiments). (f) Velocity versus time profile acquired on UV‐responsive droplets (0.5 µL) exposed to 30 s UV on–off cycles (*n* = 3 experiments).

In the design of our UV‐responsive motile droplets, we exploit the destabilizing effect of low pH on the crystalline layers. We reasoned that by starting with an oil droplet that is inert to the layer, including a photoacid generator (PAG) would, upon UV irradiation, produce a local acidic environment which disintegrates the layer and thereby drives the propulsion of the droplet. To this end, our UV‐responsive droplets consist of photoacid generator 2‐(4‐methoxystyryl)‐4,6‐bis(trichloromethyl)‐1,3,5‐triazine (33.5 mM) dissolved in mesitylene with 5 v/v% DA. As shown in Figure 2c and Movie S1, a 0.5 µL droplet that has been deposited onto a layer does not move by itself. Only when the droplet is exposed to UV from above (*t* = 0 s), the droplet starts to move upon destabilizing the layer, as evidenced by the millimetre‐wide open channel that is left behind the droplet. Interestingly, the layer is capable of regenerating over time, as evidenced by the channel formed by a UV‐responsive droplet that closes within approx. 10 min (Movie S1). In experiments with different droplet volumes, we observe that the droplet velocity decays from approx. 0.1–0.2 mm s^−1^ at the start of UV exposure to 0 mm s^−1^ over a time course of approx. 250 s (0.2 µL) to 600 s (0.5–2.0 µL), presumably as the PAG gets depleted upon exposure to UV (Figure 2d and Figures S3–S7). The levelling off of the lifetime at larger droplet sizes (0.5–2.0 µL) is indicative of slow diffusion limiting the effectiveness of PAG in the core of the droplet.

Importantly, the droplet motility is entirely dependent on UV irradiation. As shown in Figure 2e, the droplets do not move over a period of 300 s in the absence of UV. Only upon exposure to UV from *t* = 300 s onwards, the droplets start to move with average velocities up to 0.2 mm s^−1^. Importantly, we note that the inclusion of 5 v/v% DA in the droplet is critical to maintain the droplets at a standstill in the absence of UV: without DA included, a slight motility of the droplets was observed prior to UV exposure. Furthermore, the motility of the droplets can be modulated via UV on–off cycles. As shown in Figure 2f and Movie S2, in the presence of UV the droplet velocity shows a spike, whereas in the absence of UV the velocity rapidly decays. Our droplets successfully exhibited run–halt behavior over multiple cycles. With each successive cycle, the velocity progressively decreased, ultimately diminishing after approx. 600 s. This reversible response demonstrates that the droplet motility is directly governed by UV irradiation.

### 2.2 Mechanism of UV‐Responsive Droplet Movement Through Crystalline Layer

To further unravel the mechanism governing the UV‐controlled motility, we followed a UV‐responsive droplet that was stained with the Oil Red O dye in a high‐resolution camera setup. As shown in Figure 3a,c, the droplet is covered by the reflective crystalline layer directly after deposition. Intriguingly, upon exposure to UV from above at *t* = 0 s, the reflective layer on top of the droplet disappeared (*t* = 7 s). Next, as the droplet started moving, it became partly covered by the layer as indicated by the reflections from the layer appearing much less intense on the part of the droplet facing the open channel (*t* = 69 s). We hypothesize that the layer dissolves because of dissociation of the decylcarbamate building block, resulting from the acidic environment created upon UV‐activation of the PAG.

**FIGURE 3:**
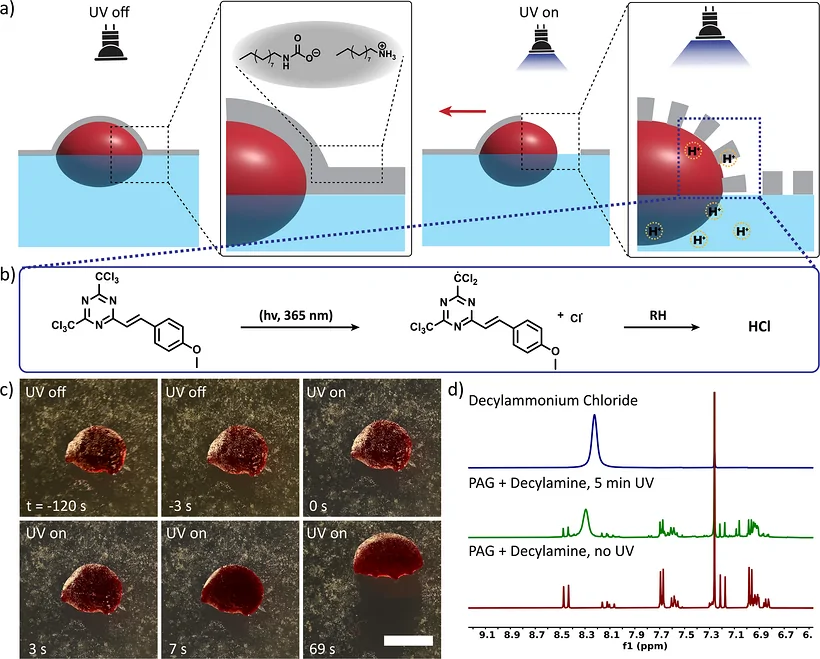
Mechanism of UV‐responsive motility of mesitylene‐based droplets through the crystalline layer. (a) Side view schematic of a UV‐responsive droplet (red) with the crystalline layer (grey) on a 3.0 mM DA aqueous solution (blue). UV irradiation (right scheme) generates a local acidic environment that protonates and locally dissolves the layer. Capillary attraction keeps the droplet in contact with the remaining layer, causing it to slip underneath and form a channel during motion. (b) Reaction scheme of UV‐induced proton generation by the photoacid generator (PAG). (c) Top view camera recordings featuring the disappearance of the reflective crystalline layer from the droplet surface upon UV exposure (from *t* = 0 s), and the asymmetric coverage of the droplet during motion, consistent with localized disintegration of the layer. The scale represents 2 mm. (d) The formation of decylammonium ions after 5 min of UV irradiation was confirmed by the appearance of characteristic peaks in the ^1^H NMR spectrum on a mixture containing PAG, *p*‐xylene and DA.

Mechanistic studies reported by Pohlers et al. [52] have shown how the PAG molecule used in our system undergoes homolytic C─Cl bond cleavage upon UV exposure, producing a highly reactive chlorine radical that abstracts hydrogen from the solvent to generate HCl. We anticipate that the generated HCl subsequently reacts with decylcarbamate, forming decylammonium chloride (Figure 3b) which leads to the disintegration of the decylammonium/carbamate‐based crystalline layer (Figure 3a). Gratifyingly, UV irradiation of a mixture of *p*‐xylene, DA and PAG demonstrates the capacity of the PAG to protonate DA, as was confirmed in ^1^H NMR spectroscopy, showing after 5 min of UV exposure a peak at 8.23 ppm that overlaps with the chemical shift of the ammonium protons of decylammonium chloride in CDCl_3_ (Figure 3d).

Together, our results corroborate the mechanism governing the motion of the UV‐responsive droplet through the crystalline layer. Dissociation of the decylcarbamate in the decylammonium/carbamate‐based layer destabilizes its crystalline structure, resulting in localized disintegration of the layer that is in contact with the droplet. Symmetry breaking occurs as the droplet detaches at one side from the layer. The droplet gets drawn through capillary attraction towards the layer that it is still in contact with, and consecutively the droplet slips under the layer to minimize its surface energy, as schematically shown in Figure 3a. This is the first step in the channel formation, converting the droplet from round to half‐moon shaped with the rear of the droplet flattened. Next, UV irradiation of the droplet sustains the continuous cycle of destabilization of the crystalline layer on top of the droplet, and propulsion of the droplet as it slips under layer areas that are still intact. Furthermore, we note that photo‐activation of the UV‐responsive droplet attracts a Marangoni flow along the channel that it has created (Movie S1), suggesting a higher surface tension close by the droplet that is in agreement with surface tension measurements showing an increase in the surface tension at the a/w interface when a UV‐responsive droplet that releases DA is exposed to UV (Figure S8). Presumably, these Marangoni flows contribute to the symmetry breaking as they push the droplet to a fresh layer. Importantly, as the droplet keeps getting into contact with fresh layer areas, continuous release of acid upon UV activation of the PAG is a prerequisite to sustain the droplet motility, as evidenced by the run–halt experiments (Figure 2f). Furthermore, in an experiment where the droplet was exposed to a UV source with a beam diameter comparable to the droplet size, we observed that the layer disintegration occurs mostly at the side of the droplet that is exposed to UV, causing the droplet to move away from the UV beam while leaving behind the open channel (Figure S9).

### 2.3 Non‐Linear Motility of Merged Droplets Through Synergistic Solvation

The photo‐control over the motility of PAG‐loaded mesitylene droplets prompted us to explore their interactions with other droplets that are immobilized within the crystalline layer. C_12_E_3_ droplets remain, upon deposition onto the layer, immobilized for an extended period of time. The layer significantly reduces the strength of outbound Marangoni flows that typically emerge around C_12_E_3_ droplets at “naked” a/w interfaces, allowing two C_12_E_3_ droplets to be placed just a few millimeters apart without affecting each other, as long as the layer keeps them separated. We reasoned that merging of a UV‐responsive mesitylene droplet can trigger the motility of these C_12_E_3_ droplets. Interestingly, we observe that in the absence of UV, the resulting merged droplet continues to move through the layer and forms a millimeter‐wide open channel, as shown schematically in Figure 4a.

**FIGURE 4:**
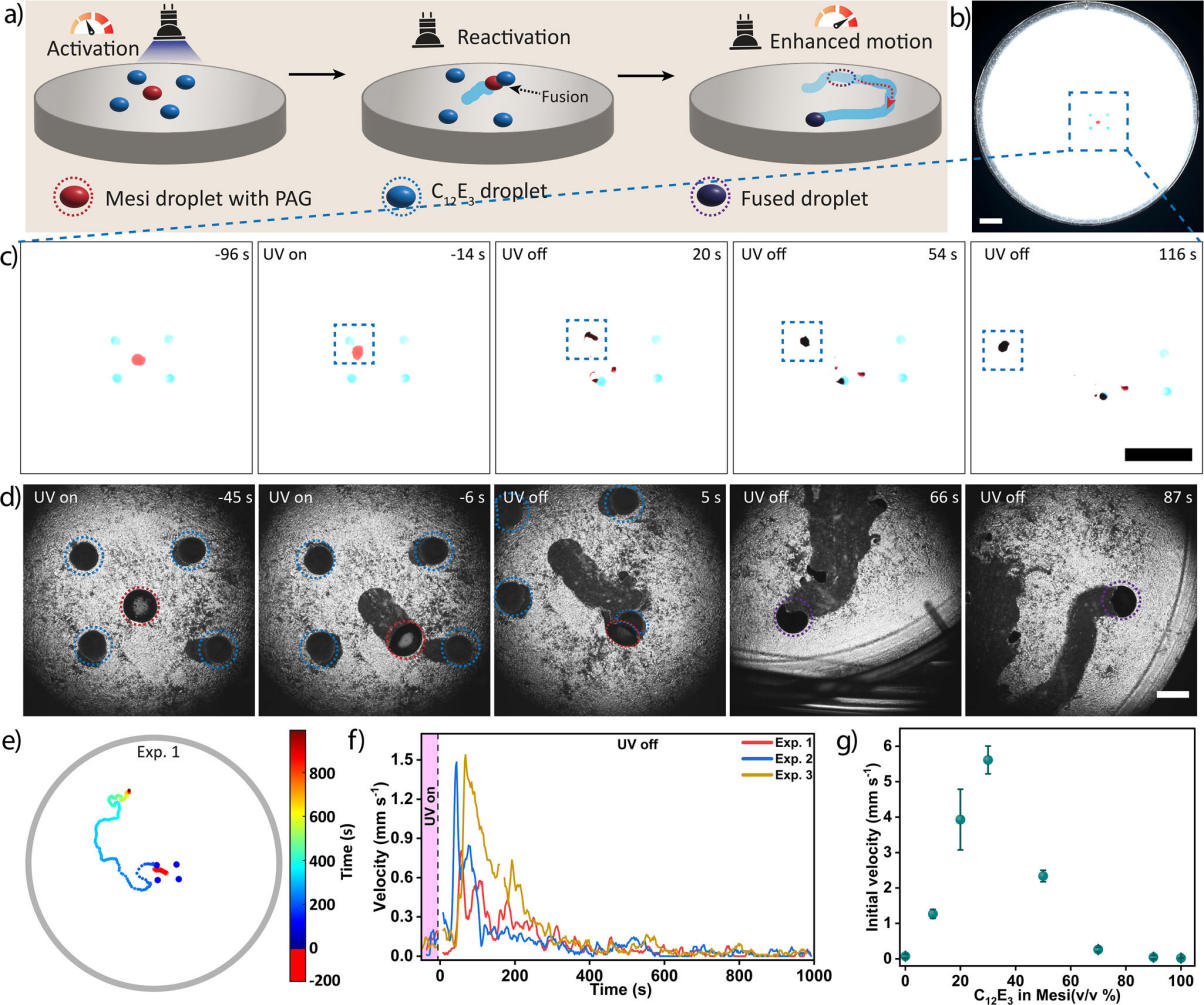
Non‐linear motility of merged droplets driven by synergistic solvation. (a) Scheme of the droplet cascade: A UV‐responsive droplet merges with an immobilized C_12_E_3_ droplet within the crystalline layer, yielding a merged droplet with an enhanced velocity that is sustained when UV is turned off. (b–d) Top view camera (b,c) and optical microscopy (d) recordings showing the interaction between one UV‐responsive droplet (1.0 µL, red) and four C_12_E_3_ droplets (0.5 µL, blue) deposited onto a crystalline layer formed on a 3.0 mM DA solution. The UV‐responsive droplet becomes motile under UV irradiation, merges with one of the C_12_E_3_ droplets, after which UV is turned off (*t* = 0 s). Next, the merged droplet (purple) exhibits an approx. 30–50 s delay before a pronounced acceleration. The scale bars represent 10 mm (b,c) and 2 mm (d), respectively. (e) Time‐resolved trajectory of the droplets, corresponding to the experiment shown in (b,c). The grey circle outlines the perimeter of the Petri dish (diameter 10 cm). (f) Velocity versus time profiles of the UV‐responsive droplet before (*t* < 0 s) and after merging with a C_12_E_3_ droplet (*t* > 0 s), for *n* = 3 independent experiments. (g) Initial velocities of droplets containing different C_12_E_3_/mesitylene ratios on a crystalline layer (*n* = 4 experiments per data point), demonstrating the effect of synergistic solvation with an optimum droplet velocity at 30 v/v% C_12_E_3_.

As shown in Figure 4b,c and Movie S3, we deposited four C_12_E_3_ droplets (0.5 µL, stained with Solvent Green 3 dye to give blue color) around one central UV‐responsive droplet (1.0 µL, red) onto the layer at the a/w interface. To prevent the droplets from colliding with the wall of the Petri dish, we continue our experiments with wide‐diameter Petri dishes (10 cm). Upon exposure to UV, the central droplet starts to move with a velocity of approx. 0.05–0.25 mm s^−1^ and typically collides with one of the C_12_E_3_ droplets, merging into a deep‐purple colored droplet. At this point, UV was turned off. Importantly, the merged droplet continues to move at relatively low velocity for a delay time of approx. 30–50 s, followed by a sudden acceleration up to 0.8–1.5 mm s^−1^ (Figure 4e,f). Subsequently, the velocity decays to 0 mm s^−1^ over a time course of approx. 600 s (*n* = 3 experiments, Figure S10).

We rationalize the sudden increase in velocity and subsequent UV‐independent motility of the merged droplet by the synergistic solvation of the building blocks of the DA‐based layer—decylammonium and ‐carbamate—by mesitylene and C_12_E_3_. As shown in Figure 4g, pure mesitylene and C_12_E_3_ droplets do –individually– not move through the layer: Their velocity upon deposition equals 0 mm s^−1^. NB decylamine is well‐miscible with both C_12_E_3_ as well as mesitylene, corroborating the effect of the decylammonium/carbamate ion pair in the system. To investigate the synergistic solvation effect in droplet motility through the layer, C_12_E_3_ was mixed with mesitylene in different ratios. We observed that C_12_E_3_/mesitylene droplets exhibit an optimum initial velocity through the layer at 30 v/v% C_12_E_3_, whereas both increasing or decreasing the C_12_E_3_ content resulted in lower initial velocities. Intriguingly, the delay in the high‐speed velocity of the droplet after merging in the experiments shown in Figure 4f can be ascribed to the mixing time—that is diffusion and convection within the droplet—that is required for the front of the droplet, which is in contact with the layer, to obtain the optimum C_12_E_3_/mesitylene ratio. We note that the concept of synergistic solvation, that is two solvents combined providing a much better solvent than what is expected based on their weighted mean, is reported for a wide range of solutes and solvent mixtures [53, 54]. Together, our results show how the combination of two different types of droplets based on non‐solvents allows triggering motion through a crystalline layer by exploiting the concept of synergistic solvation.

### 2.4 Droplet Merging Cascades in Large Fields

Having discovered a novel interaction in the merging of C_12_E_3_ and mesitylene‐based UV‐responsive droplets, mediated by the crystalline layer, our next goal was to leverage this interaction to create UV‐induced cascades in systems containing many of both types of droplets. Herein, a brief burst of UV irradiation sets UV‐responsive droplets in motion, which then merge with C_12_E_3_ droplets. Subsequently, the merged droplets accelerate and continue moving independently of UV exposure, allowing them to collide with remaining stationary droplets and sustain the cascade. However, the collision of droplets with the walls of the Petri dish typically proved to be a significant hindrance in establishing motility in large droplet swarms: droplets that collide with the wall effectively remove themselves from continued interaction with the system. To solve this problem, we sought to find a way to keep droplets from colliding with the wall of the Petri dish the crystalline layer is formed inside.

We found that droplets consisting of 60 v/v% C_12_E_3_ in DA reliably resist merging with incoming UV‐responsive droplets, and instead repel them after making contact, with the C_12_E_3_/DA droplet remaining stationary throughout the encounter. Moreover, the high viscosity of the C_12_E_3_/DA mixture allows its deposition in elongated shapes that resist deformation when embedded in the crystalline layer. Lines of this solution, which was dyed with Solvent Green 3 and deposited from underneath the crystalline layer, can effectively repel incoming droplets, acting as a boundary that restricts their movement (Figure 5b and Movie S4). We hypothesize that the high concentration of C_12_E_3_ and DA in the boundary suppresses the capacity of incoming droplets to disintegrate the layer at the side of the droplet that faces the boundary, compared to the side of the droplet not facing the boundary. The resulting imbalance causes the droplet to rapidly depart from the boundary, rather than merging to it. NB the effectiveness of the boundary is anticipated to depend both on its C_12_E_3_ content (a C_12_E_3_‐based boundary would simply merge with an incoming droplet) as well as its DA content: When the boundary contains too much DA, the crystalline layer would become unstable as was observed with pure DA droplets [6].

**FIGURE 5:**
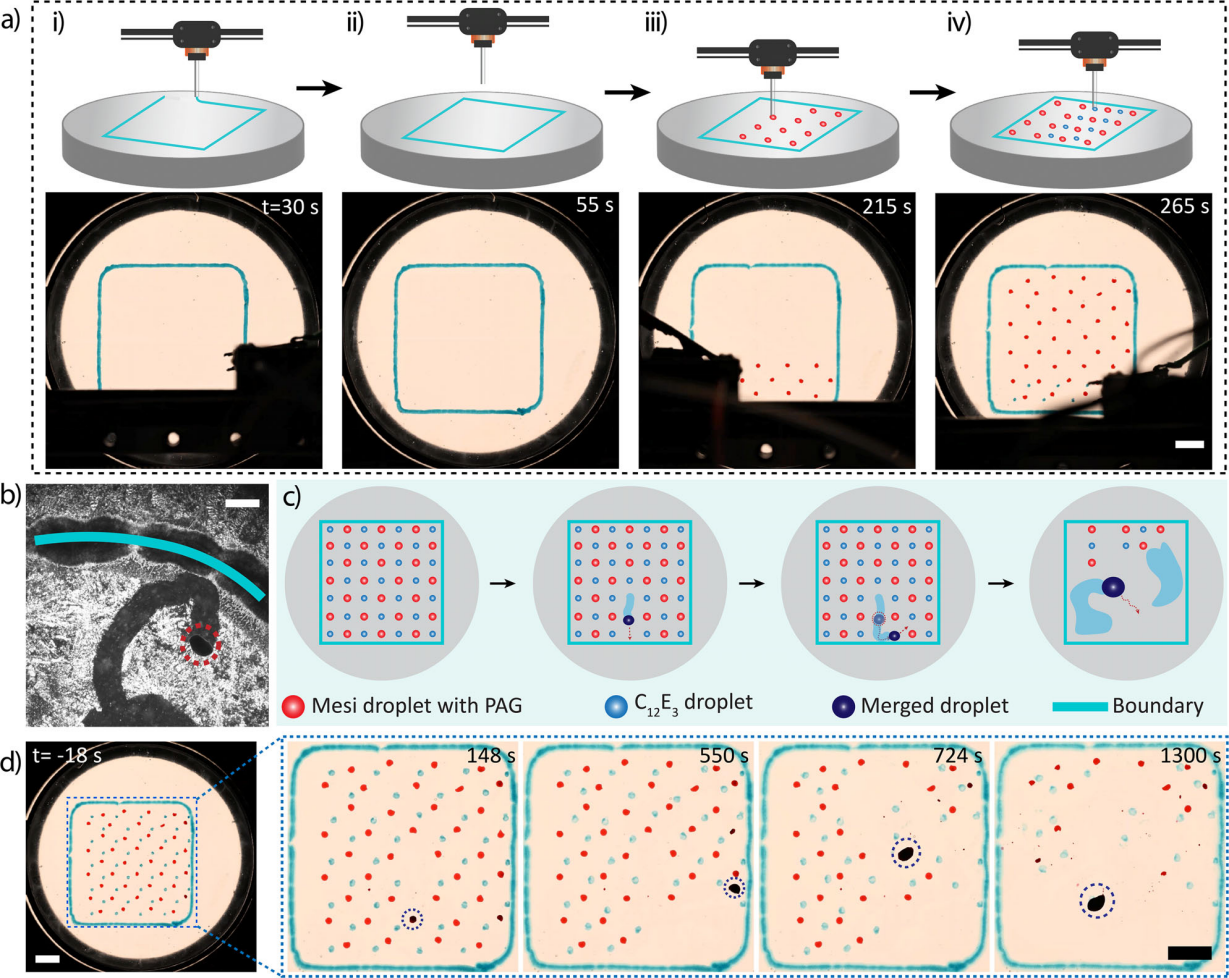
Confinement of cascading droplet motion using a C_12_E_3_/DA repellent boundary. (a) Schematic representation and camera recording of the droplet deposition robot placing both the repellent boundary mixture (60 v/v% C_12_E_3_ in DA; cyan) and individual droplets onto the crystalline layer at the a/w interface. The scale bar represents 10 mm. (b) Optical microscopy recording showing that a merged mesitylene–C_12_E_3_ droplet (indicated by red circle) is redirected upon encountering the repellent boundary (cyan arc) that has been deposited onto the layer. The scale bar represents 2 mm. (c,d) Schematic representation and camera recording of a 9 × 9 grid composed of alternating UV‐responsive droplets (0.4 µL, red) and C_12_E_3_ droplets (0.2 µL, blue) confined within a square C_12_E_3_/DA repellent boundary deposited on the layer. Merging a central red–blue pair at *t* = 0 s generates a motile hybrid droplet (dark purple) that initiates a cascade. Importantly, over the course of the experiment, the droplets are contained within the boundary, enabling a continuation of the cascade within the confined region. The scale bars represent 10 mm (left) and 5 mm (right), respectively.

Using our droplet deposition robot (Methods, Figure S13), lines of the C_12_E_3_/DA repellent boundary mixture can be deposited in square patterns at the a/w interface coated with the crystalline layer, as shown in Figure 5a. Next, we deposited within the square a 9 × 9 droplet grid consisting of 40 UV‐responsive droplets (0.4 µL, red) and 41 C_12_E_3_ droplets (0.2 µL, blue) in an alternating pattern (Figure 5a,b). Subsequently, a neighboring couple of a red and blue droplet positioned in the middle of the grid was merged by bringing them in contact with a tweezer, initiating a cascade of merging among the droplets. Indeed, the merged droplet starts moving throughout the layer, readily merging with other droplets (Figure 5c,d, Movie S4 and Figure S11). The boundary was observed to repeatedly redirect the trajectory of the droplet whenever it approached the edge of the confined area, thereby maintaining its motion within the square. Thereby, this repellent boundary strategy significantly increases the likelihood of interaction between the merged droplets and the surrounding droplets in the grid.

Finally, we investigated the collective cascade behavior of our droplet system that is initiated upon photo‐activation. As schematically shown in Figure 6a, the alternating droplet arrangement of UV‐responsive (red) and C_12_E_3_ (blue) droplets was scaled to cover the entire Petri dish, yielding a grid of 185 droplets enclosed by a circular boundary. To initiate the cascade, the middle region of the droplet grid was exposed to UV irradiation from above for 60 s. In response, only the UV‐responsive droplets located within the irradiated area became motile (Figure 6b). Over time, these droplets merged with neighbouring C_12_E_3_ droplets, and the resulting droplets continued to propagate the cascade by merging with other droplets (both red and blue) in the grid, progressively increasing their size. Importantly, we consistently observed in *n* = 9 experiments that the cascade of merging droplets continues well beyond the time span of the UV irradiation phase: In fact, right after the UV irradiation phase, only a small subset of 4 ± 4 droplets has merged, whereas over a time course of 1800 s, the number of merged droplets increases to 126 ± 49. Furthermore, in all cases, the path of the merged droplets is observed to progress outside the area of initial UV exposure (Figure 6c and Movie S5, Figure S12). We note that both the rate of droplet merging as well as the final number of merged droplet varies from experiment to experiment. However, in all cases, the time‐dependent area that has been visited by motile droplets shows a rapid increase right after UV exposure, and levels off over time as the crystalline layer gets depleted (Figure 6c).

**FIGURE 6:**
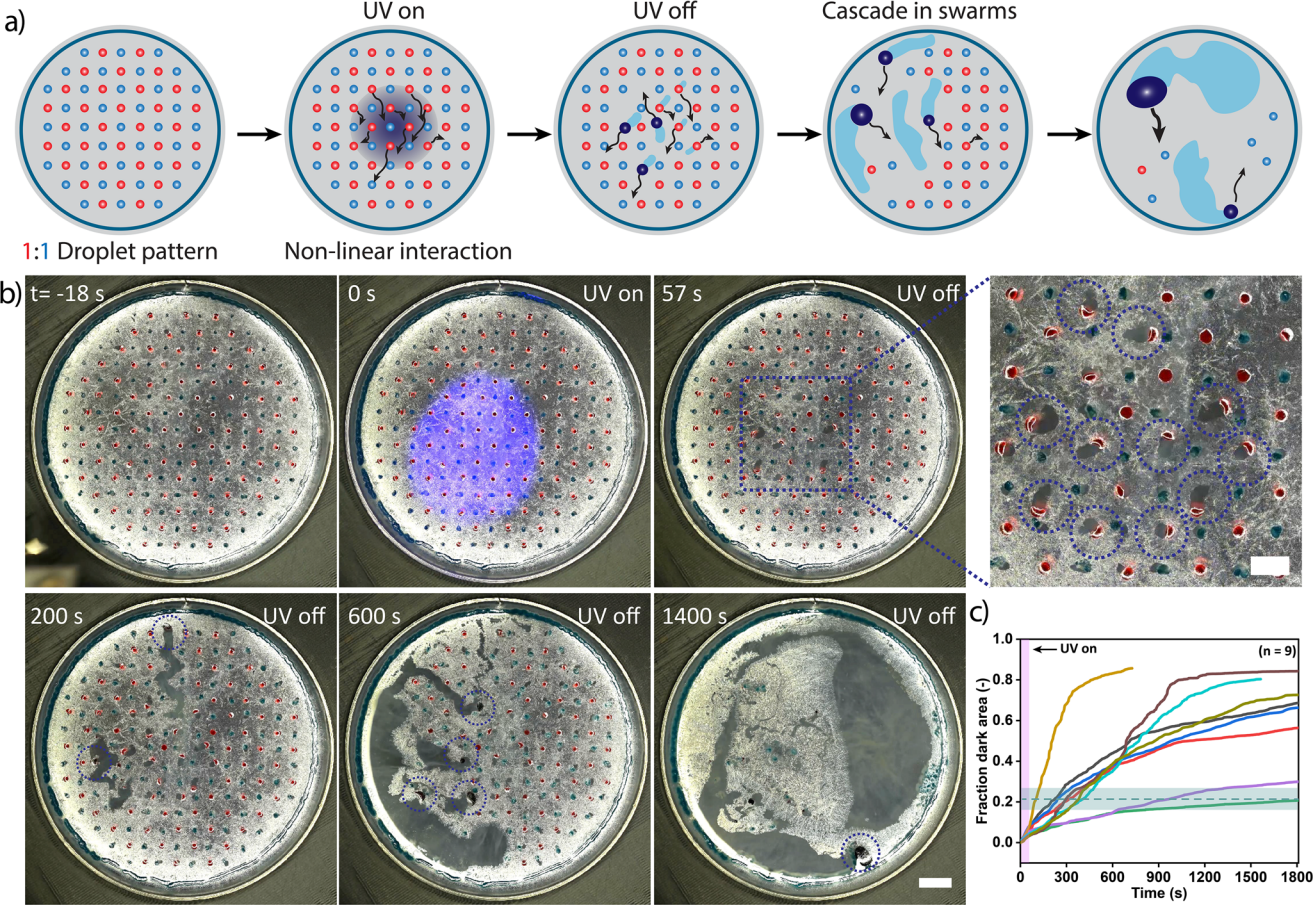
Cascading behavior in large droplet swarms. (a,b) Schematic representation and camera recording of a droplet grid covering a 10 cm wide Petri dish, consisting of 88 UV‐responsive droplets (0.4 µL, red) and 97 C_12_E_3_ droplets (0.2 µL, blue) arranged in a 1:1 alternating pattern and confined within a circular boundary (60 v/v% C_12_E_3_ in DA; cyan) deposited on the crystalline layer. At *t* = 0 s, UV irradiation focused on the center of the Petri dish activates only the UV‐responsive droplets within the exposed region (highlighted in blue). After UV is turned off (*t* = 57 s), the resulting merged droplets continue to propagate the cascade, moving beyond the initially irradiated area, merging with additional droplets, and increasing in size over time. The scale bars represent 10 mm and 5 mm (right inset), respectively. (c) Time‐dependent fraction of the dark area in the camera recordings, which are indicative of the open channels/areas in the crystalline layer, formed upon merging of motile droplets. The replicate experiments (*n* = 9) show continued merging well beyond the initial UV‐irradiation phase (60 s). The dashed horizontal line indicates the average area (± standard deviation) of the UV‐irradiated zone in the replicate experiments.

We attribute the observed variability in rate and effectiveness of the merging cascades to several factors governing the droplet–droplet interactions and the dynamics of the crystalline layer. First, effective propagation of the cascade relies on the interplay of droplet size, composition, and the non‐spherical (non‐equilibrium) shapes that droplets acquire while interacting with the crystalline layer, which affects the likelihood of a droplet to merge with another one, which in turn affects the droplet size, composition, and shape. Second, we observe that in some instances, the large droplets formed towards the end of the experiment merge with the boundary, which affects their composition and thereby their progression through the layer. Third, the availability of the layer is key to maintaining droplet motility and guiding merging events. Droplets tend to migrate toward regions where the layer is still present and avoid areas where it has already been consumed. Interestingly, we found that the layer gradually regenerates, creating a transient “memory effect”: immediately after a droplet passes, the open channel prevents it from returning, but once the layer has regrown, the same region becomes accessible again to droplets. The interplay between the layer disintegration and regeneration strongly shapes the exploratory behavior of motile merged droplets. In some cases, rapid regrowth causes droplets to circulate repeatedly within a confined area instead of exploring new regions, reducing encounters with additional droplets and thereby limiting the continuation of the cascade.

## 3 Conclusion

In summary, we present a strategy that enables cascades of chasing and merging events within motile droplet swarms. Central to our approach is a synergistic solvation mechanism: two droplet types that individually do not dissolve the decylammonium/carbamate‐based crystalline layer at an air/water interface become motile upon mixing. By combining stable crystalline layers with UV‐controlled motility of photoacid generator‐loaded mesitylene droplets, we establish a system in which selected droplets can be activated on demand, triggering merging events with otherwise immotile tri(ethylene glycol) monododecylether (C_12_E_3_) droplets. The resulting hybrid droplets display enhanced and sustained motility, allowing them to propagate through large droplet fields and initiate further merging events long after the UV stimulus is removed. Together, our concepts demonstrate how synergistic solvation and controlled dissolution of a crystalline surfactant layer can be used to orchestrate complex, multi‐step behaviors in chemically active droplet swarms.

Well‐designed systems of chemical reactions and physicochemical interactions provide versatile platforms to direct the motility of droplets as building blocks in the organization of matter at the micro‐ and macroscale. We envision that our approach opens opportunities to encode more complex logical behaviors by introducing additional droplet types, for example through orthogonal droplet combinations, multi‐step merging sequences or competitive interactions. Moreover, the crystalline ammonium/carbamate‐based surfactant layer provides a platform for orchestrating the interactions among such droplets –potentially even at microscale– enabling experiments that begin from well‐defined initial arrangements and ultimately guide the assembly of multi‐droplet structures as material‐based outcomes. Finally, extending the principles of this work to three‐dimensional interfaces of droplets embedded within crystalline surfactant coatings could offer new possibilities in the formulation of out‐of‐equilibrium emulsions, such as Janus‐type particles.

## 4 Methods

### 4.1 Materials

All chemicals and materials were used as received: triethylene glycol monododecyl ether (C_12_E_3_, ≥95%) and 2‐(4‐methoxystyryl)‐4,6‐bis(trichloromethyl)‐1,3,5‐triazine (≥98%) were purchased from TCI chemicals. Decylamine (≥95%), 1,3,5‐trimethylbenzene (mesitylene), deuterated chloroform (CDCl_3_, ≥99.9%), dimethyl sulfoxide‐*d*_6_ (DMSO‐*d*_6_, ≥ 99.9%), Solvent Green 3 (≥95%), *p*‐xylene (≥99%) and Oil Red O were purchased from Sigma‐Aldrich. Hydrochloric acid (2 M) was purchased from Fisher Scientific. Milli‐Q water (18.2 MΩ.cm) was used throughout the experiments.

### 4.2 Crystalline Layer Formation

To prepare the crystalline layer at the air/water interface, we used an aqueous solution of 3.0 mM DA. Experiments were conducted using 35 mm and 100 mm diameter Petri dishes (Falcon), filled with 5.5–6 mL and 60 mL of the DA solution, respectively. The layer was typically formed within 5 min, after which complete coverage of the a/w interface was verified before droplet deposition. All experiments were performed at room temperature.

### 4.3 Droplet Deposition

The droplets were deposited at the crystalline layer with a Gilson Pipetman P2 pipette (manually), unless it is mentioned that they are deposited in larger groups with the deposition robot (Figures 5 and 6, *vide infra*).

### 4.4 Optical Microscopy

Optical microscopy images and videos were recorded with an Olympus IX73 dark‐field inverted microscope equipped with a Point Grey Grasshopper3 camera. A 1.25× objective was used; brightness and condenser filters were adjusted appropriately. The videos were recorded at 1 fps unless stated otherwise. In the optical microscopy experiments, 35 mm Petri dishes were used.

### 4.5 UV Irradiation

Three different UV sources were used. UV irradiation of a subset of the 185 droplets deposited in a 100 mm diameter Petri dish was performed with an OmniCure S1000 UV source (360 nm), mounted 15 cm above the Petri dish. The intensity was set to 50%, and the aperture of the UV guide was set such that a UV spot of approx. 35 mm diameter was created with an intensity of approx. 34 mW cm^−2^ (measured with a Thorlabs PM400A power meter with a 5170 C detector). UV irradiation of entire diameter Petri dish areas (Figures 2, 3, 4) was performed with a Kessil PR160L Gen2 UV lamp (370 nm LED, 60.2 mW cm^−2^), mounted 25 cm above the Petri dish. A handheld Tank007 UV flashlight with a modified lens to focus the UV beam (365 nm LED, 75 mW cm^−2^, beam size 1 × 1 mm^2^) was used for partial irradiation of droplets (Figure S9).

### 4.6 Probing the UV‐Responsive Droplet Velocity Over Time

To determine the velocity of a UV‐responsive droplet over time (Figures 2d–f and 4), a droplet was deposited on the crystalline layer in the middle of a 100 mm Petri dish and exposed to UV (Kessil UV lamp), while being recorded with a mirrorless camera (Nikon Z5) and macro lens (Laowa 100 mm f/2.8), mounted above the Petri dish (25 cm). The Petri dish was illuminated with a LED light panel (Viltrox VL‐200T, colour temperature 5600 K) from the bottom. The camera settings were as follows: ISO: 100, shutter speed1/40 s, aperture f22, capture rate: 0.5 fps for Figures 2e, 4, and  1 fps for Figure 2d,f. The UV‐responsive droplets are stained with Oil Red O (5 mg mL^−1^); the C_12_E_3_ droplets with Solvent Green 3 (3 mg mL^−1^). During the experiments, the entire setup is enclosed in a PVC box to prevent ambient air‐flows disturbing the floating droplets at the a/w interface.

### 4.7 High‐resolution Camera Recording of UV‐Responsive Droplet

The high resolution camera recordings of the interaction of the UV‐responsive droplet with the crystalline layer under UV exposure (Figure 3c) was performed with a mirrorless camera (Nikon Z5) and macro lens (Laowa 100 mm f/2.8), and the Kessil UV lamp.

### 4.8 IR‐ATR Spectroscopy on Crystalline Layer and Decylamine

The crystalline layer was isolated from the aqueous DA solution and directly used to analyze in IR‐ATR. Similarly, pure decylamine (5 µL) was directly used to analyze in IR‐ATR. During the measurement we used the (%) transmittance vs wavenumber (cm^−1^) method within the range of 500 to 4000 cm^−1^. The data resolution was 4 cm^−1^ and 20 scans per experiment; The experiments were performed at 293 K. IR spectra were recorded with a Shimadzu spirit IR‐ATR instrument.

### 4.9 ^1^H NMR Spectroscopy on Protonation of Decylamine

2‐(4‐methoxystyryl)‐4,6‐bis(trichloromethyl)‐1,3,5‐triazine (45 mg) was dissolved in a mixture of *p*‐xylene (3.0 mL) and decylamine (50 µL) in a 12 mm wide test tube. A magnetic stir bar was added and the test tube was irradiated with the Kessil UV lamp (370 nm) at 6 cm distance upon stirring. Aliquots (0.3 mL) were taken after 0 and 5 min of UV irradiation. The aliquots were evaporated under reduced pressure, dissolved in CDCl_3_ (500 µL) and analysed by ^1^H NMR. Additionally, decylamine (500 µL, 2.5 mmol) was mixed with aqueous HCl (2 M, 700 µL). The mixture was vigorously shaken, then desiccated to yield quantitative decylammonium chloride, and analyzed by ^1^H NMR (CDCl_3_). NMR spectra were recorded with an Agilent 400 MHz INOVA spectrometer, equipped with a dual‐channel inverse probe and a ^1^H frequency of 400.13 MHz. The experiments were performed at 293 K, tetramethylsilane was used as an internal reference.

### 4.10 ^1^H NMR Spectroscopy on Crystalline Layer and Decylamine

The crystalline layer was isolated from the aqueous DA solution, dissolved in DMSO‐*d*_6_ (500 µL) and analyzed by ^1^H NMR. Similarly, pure decylamine (20 uL) was dissolved in DMSO‐*d*_6_ (500 µL) and analyzed by ^1^H NMR. The experiments were performed at 293 K, tetramethylsilane was used as an internal reference.

### 4.11 Surface Tension Measurements of UV‐Responsive Droplet on MilliQ Water

To measure the surface tension of the aqueous solutions, we used a force tensiometer (Biolin Scientific Sigma 701) with a platinum Wilhelmy plate (wetted length 39.2 mm). Before each measurement, the plate was rinsed with ethanol and heated with a flame torch until it became red hot. For every experiment, 5 mL MilliQ water was added to a polystyrene Petri dish (Falcon, 35 mm) and a UV‐responsive droplet (2.0 µL) was deposited carefully at the a/w interface. The UV‐source (Kessil) was switched on after approx. 2 min. The Wilhelmy plate was dipped at the a/w interface, such that the solution wetted the plate and formed a meniscus.

### 4.12 Determining Initial Velocities of C_12_E_3_/Mesitylene Droplets

For the experiment shown in Figure 4g, we deposited a 0.2 µL droplet of the respective C_12_E_3_/mesitylene mixture on the crystalline layer in a 35 mm Petri dish with a Gilson Pipetman P2 pipette. The droplet trajectory was recorded in optical microscopy and measured by using the segmented line tool in *ImageJ*. Next, the velocity was determined via the path length of the trajectory and the time difference between the first and the last frame of the respective trajectory (with faster droplets being measured over a shorter time span).

### 4.13 Droplet Deposition Robot Setup

For accurate deposition of the droplets, a custom‐made droplet deposition robot developed by *Labm8* (labm8.io) was used, in analogy to the system we reported in [35]. The robot comprises a linear motion gantry, deposition head, syringe pumps and a controlling unit (Labm8, M8001.1), as shown in Figure S13. The deposition head is equipped with two needles (Fisnar, inner diameter 0.20 mm; 27 Ga), each connected with PTFE tubing with 0.81 mm inner diameter to a Hamilton Gastight 1 mL syringe. The syringes are actuated by motorized axes, that is syringe pumps (Labm8, M8003.1). The gantry, moving the deposition head, comprises four stepper motors to move in the z‐direction, two stepper motors to move in the y‐direction, and one to move in the x‐direction using a linear belt driver. To deposit different droplet solutions, the depositor head can be equipped with two needles connected to separate syringes. The needles are both fixed to the head, so they have been spaced 100 mm apart to ensure that if one deposits a droplet at any place within the 100 mm Petri dish, the other is not touching the a/w interface as well. The needle holders can easily be replaced, allowing for deposition of three different liquids upon insertion of another needle connected to a third syringe.

The stepper motor and syringe pumps are controlled by a Duet Wi‐Fi which executes G‐code scripts. All motors were calibrated, so that the imposed actuation in steps can be converted to movement in millimeters or volume in microliters. The motorized axes are mounted onto an aluminum base plate. The entire setup is mounted on an anti‐vibration optical table and levelled using adjustable legs. A Petri dish (100 mm) is placed on a hole in the base plate, through which a LED backlight (Jupio PowerLED 288, brightness 80%, color temperature 4000 K) can illuminate from the bottom. To visualize the crystalline layer during camera recordings (Figure 6c and Figure S11), a custom‐made 3D‐printed LED ring holder (17 cm diameter) equipped with an LED light (LED strip COB, Neutral white light, 4000k) is positioned above the sample. The droplets are recorded with a mirrorless camera (Nikon Z5) with a macro lens (Laowa 2x Macro 100 mm f/2.8) positioned above the Petri dish; the UV light source (OmniCure S1000) was mounted above the Petri dish, just out of view of the camera. During the experiments, the entire setup is enclosed in a PVC box to prevent ambient air‐flows disturbing the floating droplets at the a/w interface.

### 4.14 Automated Deposition of Droplets

The deposition of a droplet starts by moving the respective needle to the desired location and probing the height of the surface of the medium by lowering the deposition head until the needle touches the surface. A 2‐wire analog electrical probing circuit connected to the stainless‐steel needle is used and a copper wire positioned inside the aqueous solution. When the needle and the surface of the aqueous solution come into contact, a signal is registered (above a set threshold), which is interpreted by the robot to stop motion in the z‐direction. We note that the conductivity of the DA solutions is sufficient for the probe to detect an over‐threshold event. Next, the deposition head is raised 1 mm, and the droplet solution is pushed out by the syringe pump, creating a droplet which adheres to the side of the needle. The deposition head is then lowered at 7 mm s^−1^ until the tip of the needle is at the previously determined height of the a/w interface, after which it is immediately raised again at the same speed.

### 4.15 Depositing Repellent Boundary and Droplet Grids With the Droplet Deposition Robot

The experiments shown in Figures 5 and 6 are performed on a 100 mm Petri dish with 60 mL 3.0 mM DA solution. After the formation of the crystalline layer, first the repellent boundary solution of 60 v/v% C_12_E_3_ in DA, dyed with 3 mg mL^−1^ Solvent Green 3, was deposited by probing the surface upon moving the respective needle downward, and then further lowering the deposition head 4 mm. The head was then moved in a square or circular path at a speed of 4 mm s^−1^, depositing the repellent boundary solution at a rate of 0.33 µL mm^−1^. Next, the mesitylene‐based and C_12_E_3_ droplets were deposited—one category after the other. The droplets are deposited in a staggered pattern, starting at the left side of the top row, moving to the right, and then at the next row moving from right to left, continuing in a zigzag fashion. The height of the a/w interface is only probed once at the location of the first droplet of the respective category. After depositing of a droplet, the deposition head is moved along the x‐ and y‐axes at 20 mm s^−1^ to the location of the next droplet deposition. In the experiments shown in Figures 5a,d and 6c, the distance between two consecutive droplets is 5 mm. Altogether, the deposition of the circular repellent boundary and 185 droplets is completed within approx. 5 min. After the completion of the deposition, the deposition head is moved out of view of the camera.

### 4.16 Matlab Analysis of Motile Droplets

The camera recordings involved in the experiments of Figure 2d–f were analyzed using a custom‐made MATLAB script that used background subtraction (using a reference frame that was acquired prior to deposition of the droplet) followed by *image binarization* (*im2bw*) to isolate the droplet in each frame, and then determined the x‐ and y‐position of the droplet by averaging the coordinates of white pixels. The velocity of the droplet in any frame *n* was calculated as the distance between the positions of the droplet in frame *n* and frame *n+1* divided by one second. In the analysis of the camera recordings in the experiments in Figure 4, a custom‐made MATLAB script was used to isolate the separate RGB‐channels (*imsplit*). The difference between the Red‐ and Blue‐channel for each frame was used, followed by *image binarization* to probe the UV‐responsive droplet during the UV exposure period. The position of the dark‐purple merged droplet that was formed after UV exposure was probed via the inverted grey scale image of each frame (obtained via *rgb2gray*), applying background subtraction (using a frame that was acquired prior to deposition of the droplets) and subsequently applying a Gaussian filter (*imgaussfilt*) on the frame. The maximum in the obtained frame provided the location of the mass middle point of the largest droplet. The time‐dependent velocities of the respective droplets that are probed in the UV‐on and UV‐off periods were calculated via the displacement of the mass middle point with respect to its location in the previous frame. The locations of the blue droplets were determined via the difference between the Blue‐ and Red‐channel, followed by a Gaussian filter (*imgaussfilt*) and detection of local maxima (*imregionalmax*). The perimeter of the Petri dish was detected via the function *imfindcircles*. For the analysis of the movies in Figure S12, the area of the UV‐irradiated zone was determined via *image binarization* on a Blue‐channel frame acquired during UV exposure (threshold > 200); the boundary of the UV‐irradiated zone was determined via the *bwboundaries* function. To determine the pixel locations that have acquired a dark appearance (e.g. due to the motility of merged purple droplets) over the course of the experiment, a custom‐made MATLAB script assessed in every frame number *i* for every grey‐scaled pixel (obtained via *rgb2gray*) with position (x,y) whether it has a lower value compared to the reference frame: If *Frame_ref*(x,y)—*Frame_i*(x,y) > 10, then in the storage matrix *Track*, the corresponding pixel (x,y) is set from 255 to 0. This implies that the number of black pixels (i.e., *Track*(x,y) = 0) in the storage matrix increase over time. By performing a summation of the black pixels in *Track* for ever frame number *i*, the fraction of the black area can be assessed vs time. We note that in the time frame where UV is applied, *Frame_ref* is the first frame acquired upon UV exposure, whereas in the time frame when UV is turned off, *Frame_ref* is the first frame acquired when UV is turned off.

## Author Contributions

**Priyanshu Singh**: investigation, writing − review and editing, writing − original draft, conceptualization, methodology, validation. **Roël M. Vreuls**: investigation, writing − review and editing, writing − original draft, conceptualization, methodology, validation. **Gerco W. A. Meeussen**: investigation, conceptualization. **Peter A. Korevaar**: conceptualization, investigation, funding acquisition, writing − review and editing, supervision.

## Conflicts of Interest

The authors declare no conflicts of interests.

## Supporting information




**Supporting File 1**: smll75101‐sup‐0001‐SuppMat.pdf.

**Supporting File 2**: smll75101‐sup‐0002‐MovieS1.mp4.

**Supporting File 3**: smll75101‐sup‐0003‐MovieS2.mp4.

**Supporting File 4**: smll75101‐sup‐0004‐MovieS3.mp4.

**Supporting File 5**: smll75101‐sup‐0005‐MovieS4.mp4.

**Supporting File 6**: smll75101‐sup‐0006‐MovieS5.mp4.

## Data Availability

The data that support the findings of this study are available in the supplementary material of this article.
