# Supplementary material for: Photo‐Activated Merging Cascades in Droplet Swarms Upon Synergistic Solvation of Surfactants
Source: Small. 2026 Aug 13;22(57):e75101. doi: 10.1002/smll.75101 (PMC13659792; doi:10.1002/smll.75101)
Supplement: Supplementary file 1 [file SMLL-22-e75101-s003.pdf]

Supporting Information

Photo-Activated Merging Cascades in Droplet Swarms upon Synergistic Solvation of Surfactants.

Priyanshu Singh#, Roël M. Vreuls#, Gerco W.A. Meeussen & Peter A. Korevaar*

Institute for Molecules and Materials, Radboud University, Heyendaalseweg 135, Nijmegen 6525 AJ, The Netherlands *E-mail: p.korevaar@science.ru.nl*
# These authors contributed equally to this work.

Table of Contents

Supporting Figures

Figure S1: IR transmittance spectra of crystalline layer and decylamine.

Figure S2: ^{1}H NMR spectra of crystalline layer and decylamine in DMSO-d_6

Figure S3: Velocity vs time profiles acquired on UV-responsive droplets.

Figure S4: Time-dependent positioning traces on UV-responsive droplets (0.2 µL).

Figure S5: Time-dependent positioning traces on UV-responsive droplets (0.5 µL).

Figure S6: Time-dependent positioning traces on UV-responsive droplets (1.0 µL).

Figure S7: Time-dependent positioning traces on UV-responsive droplets (2.0 µL).

Figure S8. Surface tension measurements probing the activity of UV-responsive droplets

Figure S9: Movement of UV-responsive droplet exposed to handheld UV flashlight.

Figure S10: Time-resolved positioning traces - replicate experiments of Figure 4b-e.

Figure S11: Camera recordings to visualize the crystalline layer around the repellent boundary.

Figure S12: Image analysis of the camera recordings on 185-membered droplet grids.

Figure S13: Droplet deposition robot setup.

Description of Movies S1 – S5.

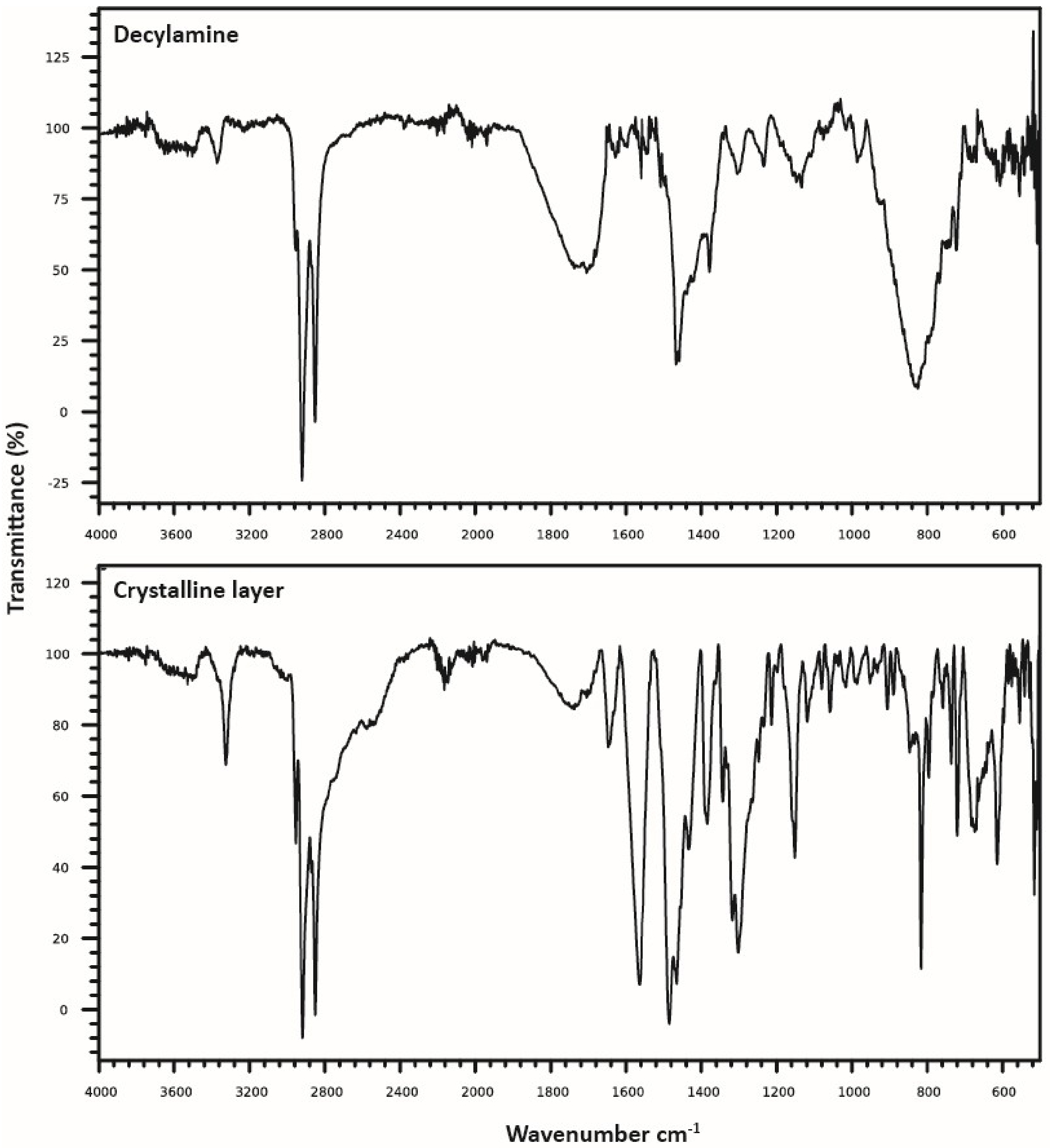

Figure S1. IR transmittance spectra of decylamine and the crystalline layer (acquired upon isolation from the a/w interface of an aqueous decylamine solution). The IR spectrum of the crystalline layer shows characteristic bands indicative of a carbamate at 1560 cm^{-1} and 1485 cm^{-1}, corresponding to the asymmetric and symmetric stretching vibrations of the $O=C=O^-$ group, respectively, as well as a band at 1647 cm^{-1} corresponding to the asymmetric vibration of ^+N-H. Importantly, these characteristic carbamate bands are absent in the IR spectrum of decylamine.

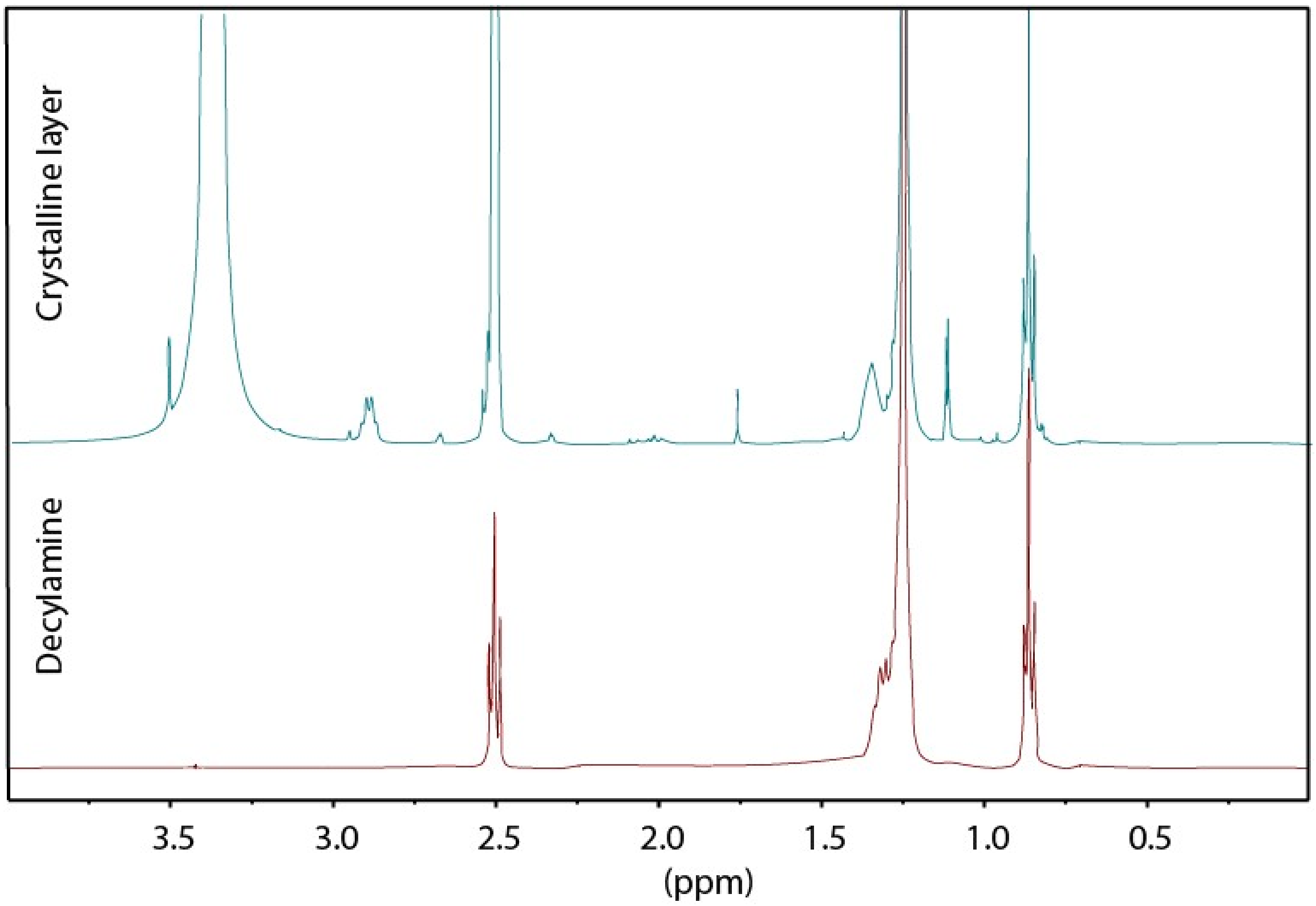

Figure S2. ^{1}H NMR spectra of the crystalline layer (acquired upon isolation from the a/w interface of an aqueous decylamine solution) and decylamine (solvent DMSO-d_6). A new peak appears at 2.85 ppm in the spectrum of the crystalline layer, corresponding to the downfield chemical shift of the methylene group at the α-position of the amide upon carbamate formation. This peak is absent in the spectrum of pure decylamine. We note that the solvent peak of DMSO at 2.5 ppm is strongly apparent is the spectrum of the crystalline layer, while absent in the spectrum of decylamine. We ascribe this difference to the decylamine-DMSO-d_6 solution being more concentrated than the solution of the isolated crystalline layer in DMSO-d_6.

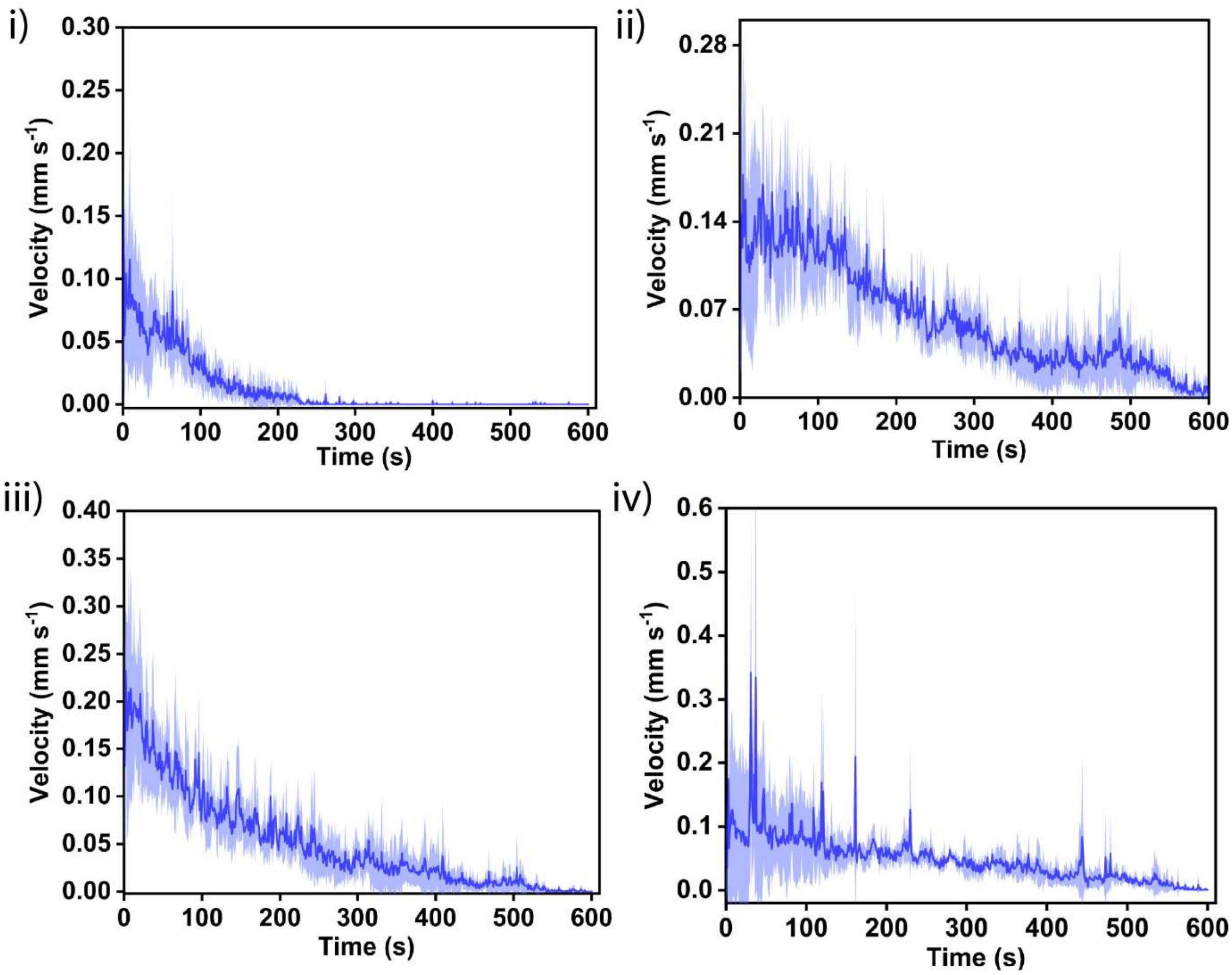

Figure S3. Velocity vs time profiles acquired on UV-responsive droplets after deposition on a crystalline layer in a 100 mm Petri dish (3.0 mM DA, 60 mL). The graphs correspond to UV-responsive droplets with different volumes: i) 0.2 µL, ii) 0.5 µL, iii) 1.0 µL and iv) 2.0 µL. Each graph is based on n = 4 replicate experiments. The averages (in bold blue) are plotted in Figure 2d of the main text.

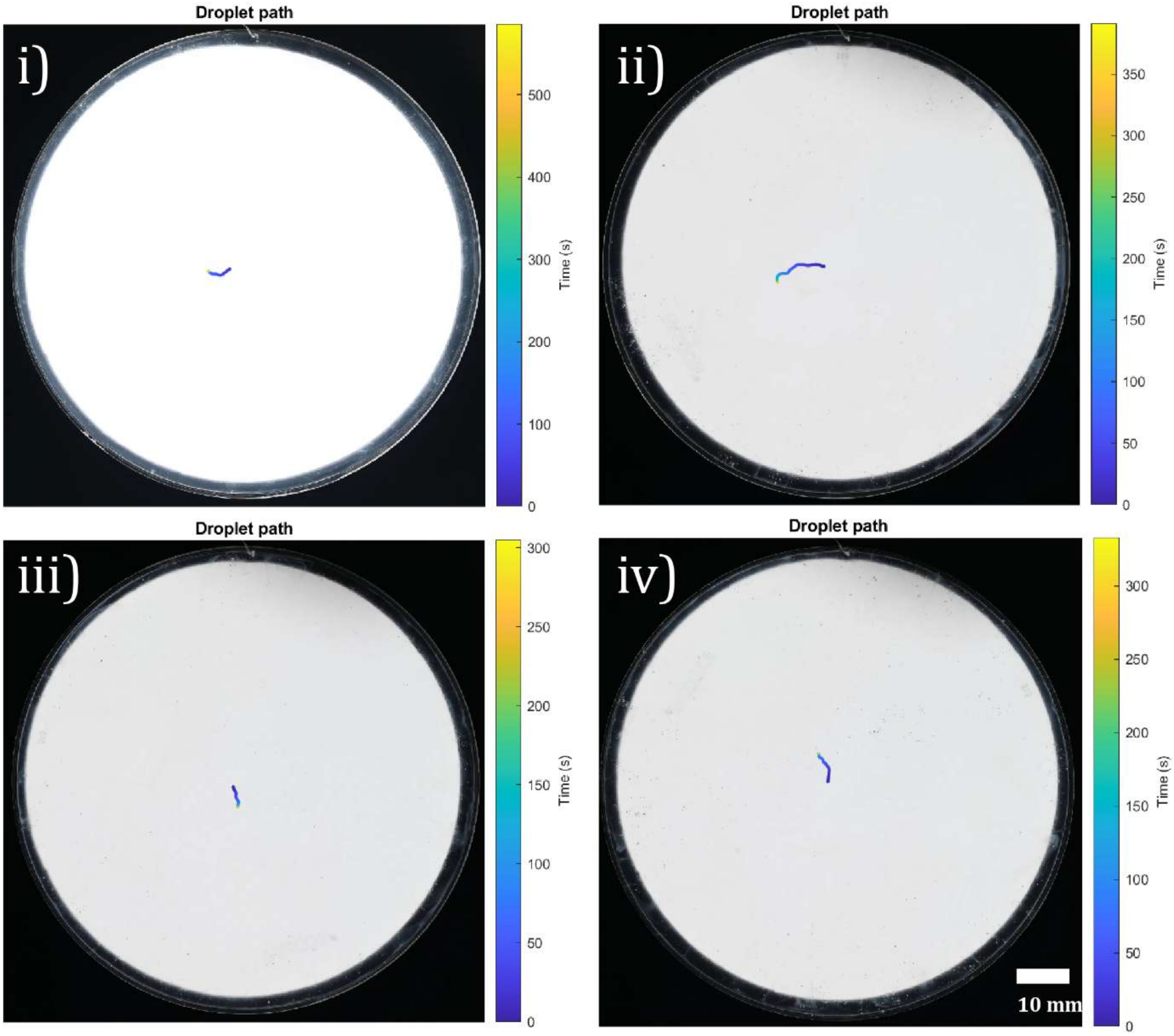

Figure S4. Time-dependent positioning traces acquired on camera recordings of four replicate experiments, where the path of an individual UV-responsive droplet (0.2 µL) is tracked over time. The underlying images represent the Petri dish area (100 mm diameter). These 4 experiments correspond to the graph presented in Figure S3-i.

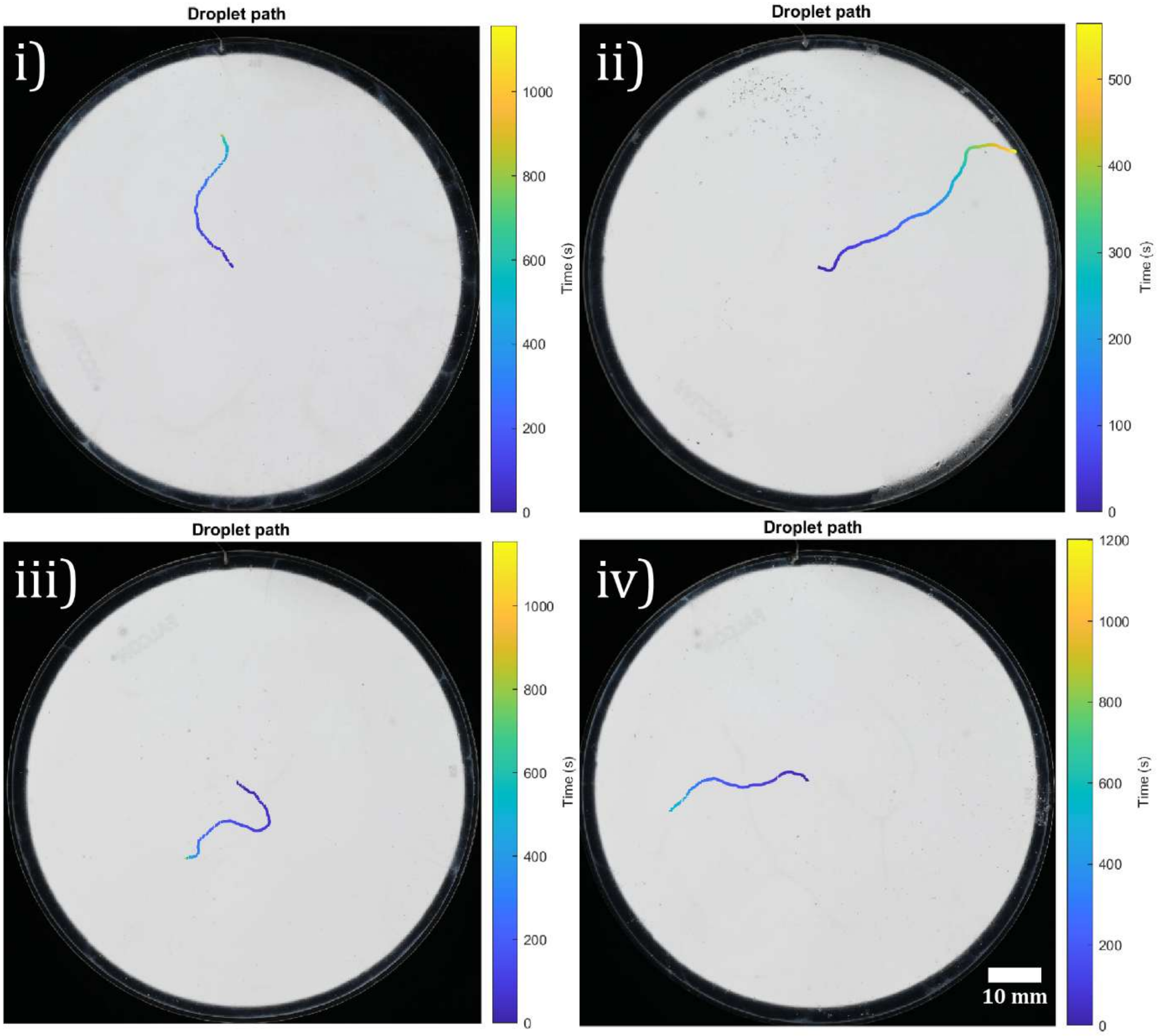

Figure S5. Time-dependent positioning traces acquired on camera recordings of four replicate experiments, where the path of an individual UV-responsive droplet (0.5 µL) is tracked over time. The underlying images represent the Petri dish area (100 mm diameter). These 4 experiments correspond to the graph presented in Figure S3-ii.

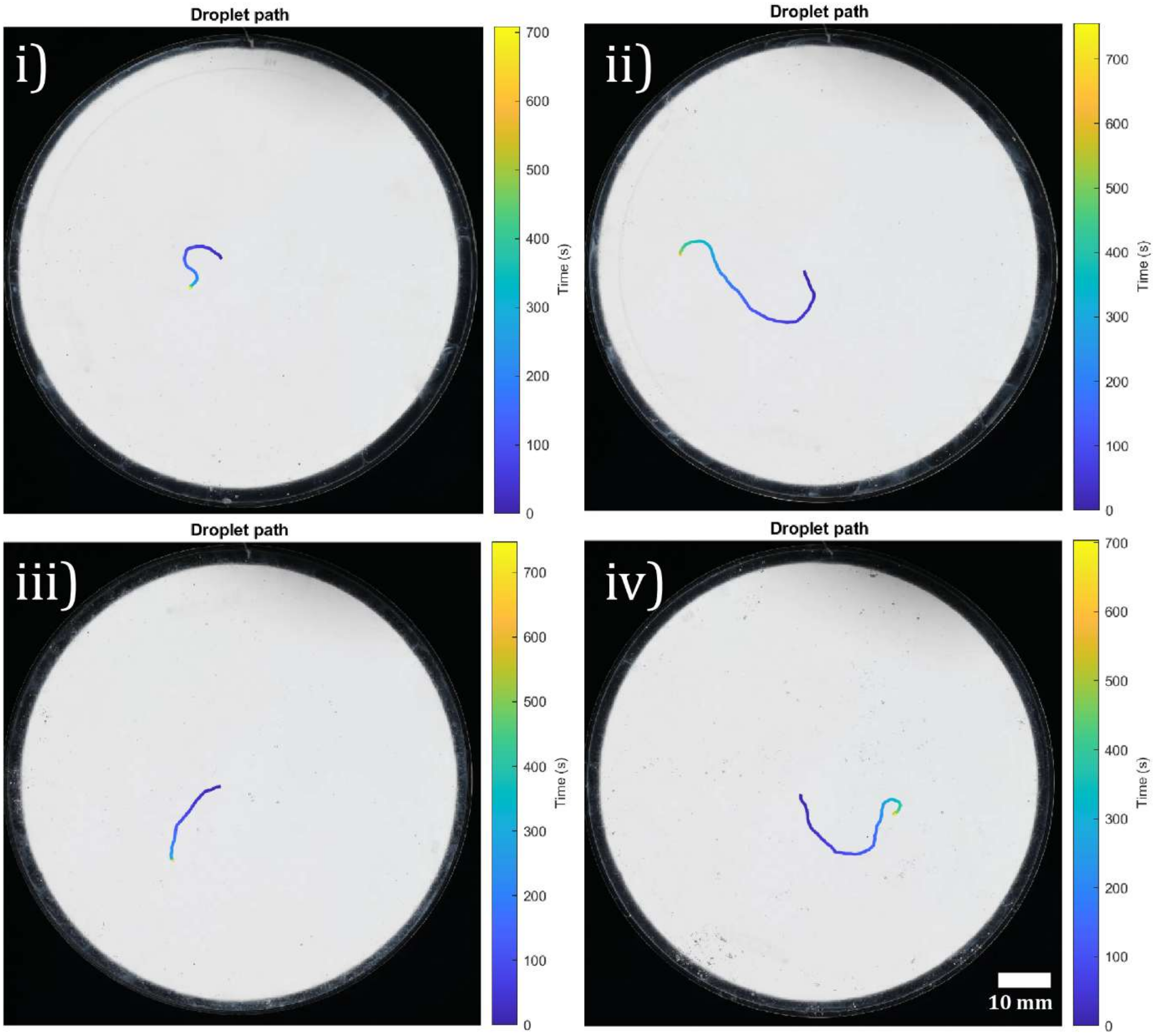

Figure S6. Time-dependent positioning traces acquired on camera recordings of four replicate experiments, where the path of an individual UV-responsive droplet (1.0 µL) is tracked over time. The underlying images represent the Petri dish area (100 mm diameter). These 4 experiments correspond to the graph presented in Figure S3-iii.

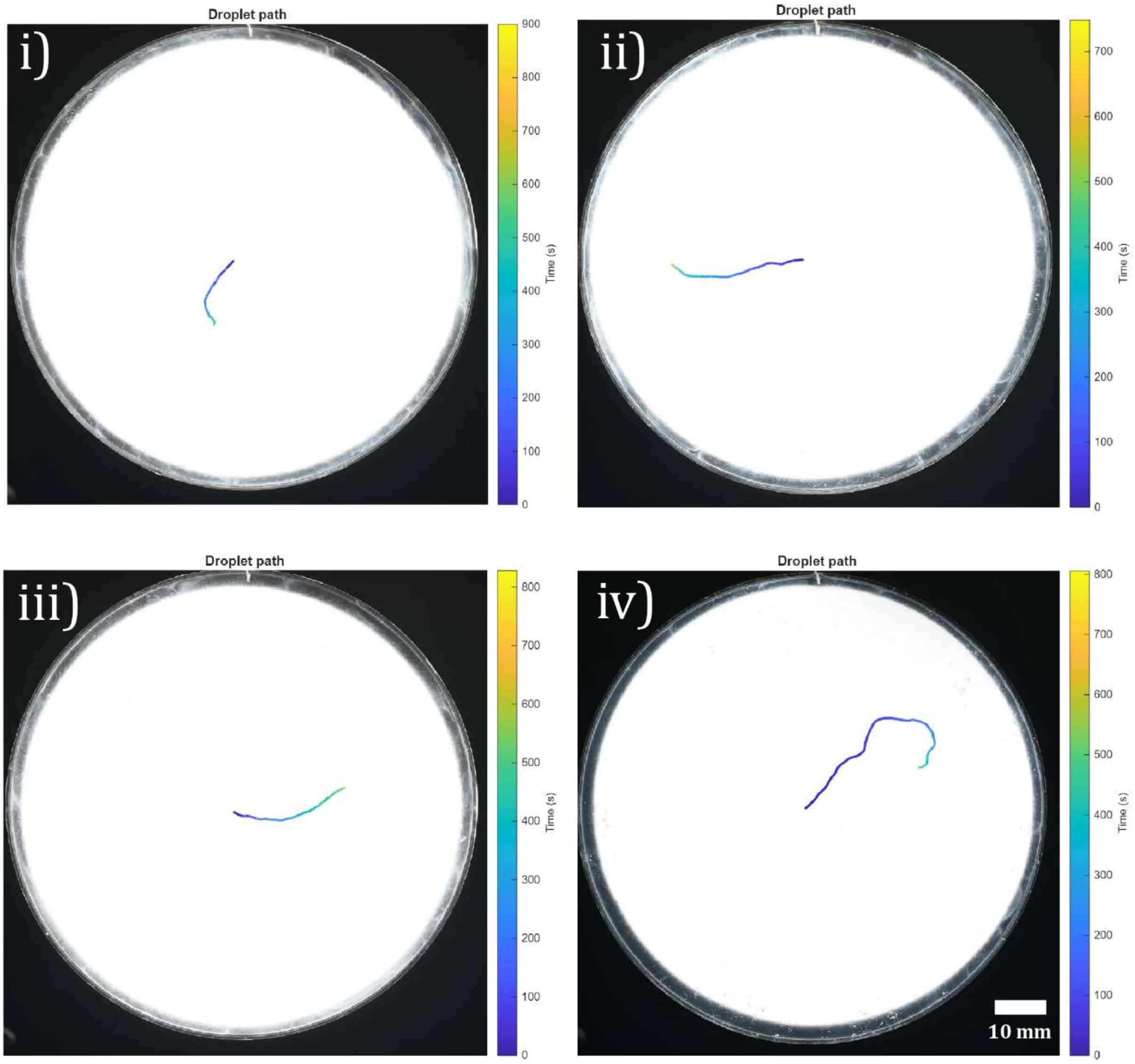

Figure S7. Time-dependent positioning traces acquired on camera recordings of four replicate experiments, where the path of an individual UV-responsive droplet (2.0 μL) is tracked over time. The underlying images represent the Petri dish area (100 mm diameter). These 4 experiments correspond to the graph presented in Figure S3-iv.

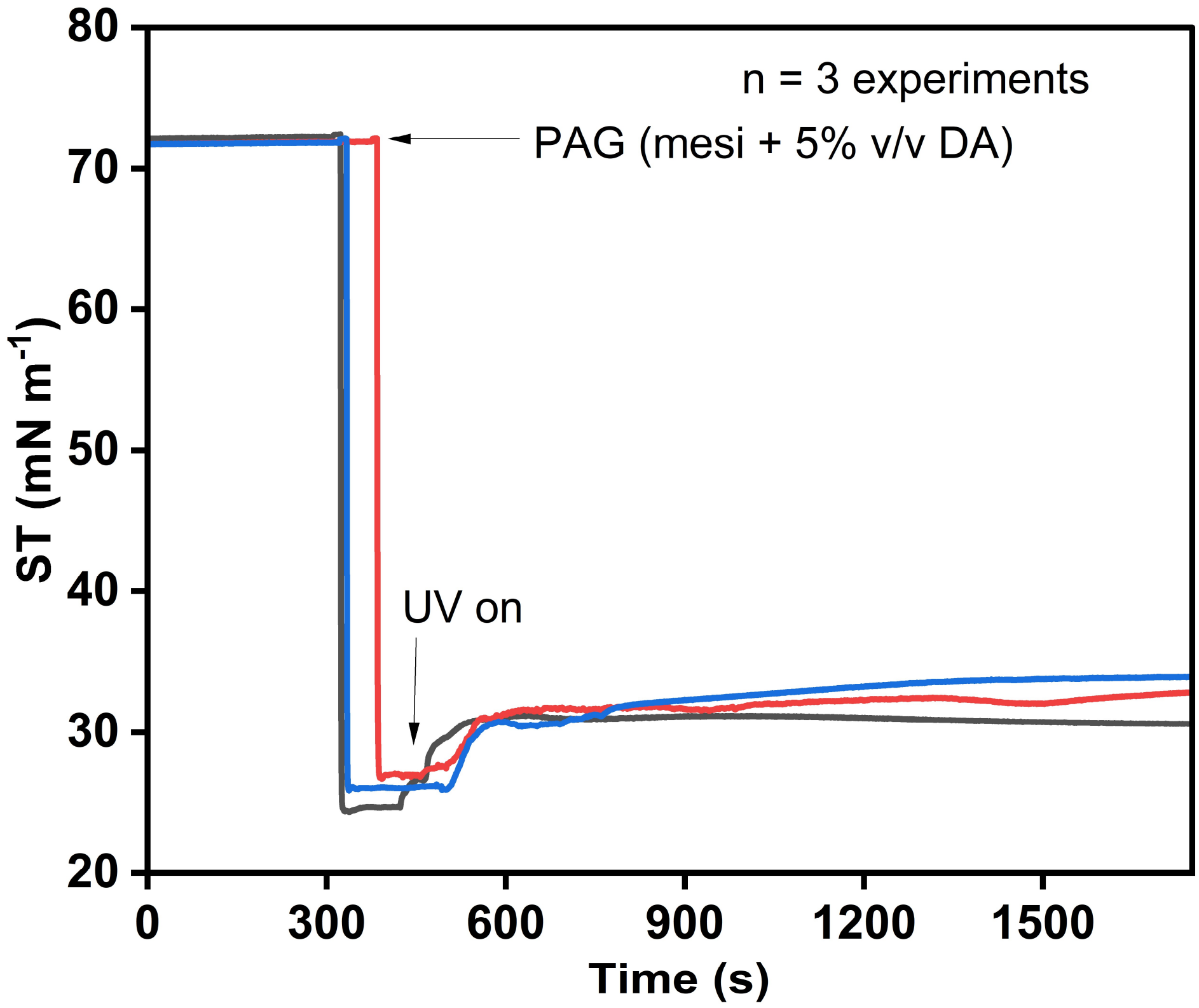

Figure S8. Surface tension (ST) measurements probing the activity of UV-responsive droplets (2.0 μL), that are deposited at the a/w interface of MilliQ at approx. 300 s, resulting in a decrease in ST. Upon UV exposure (after approx. 2 min), the ST increases.

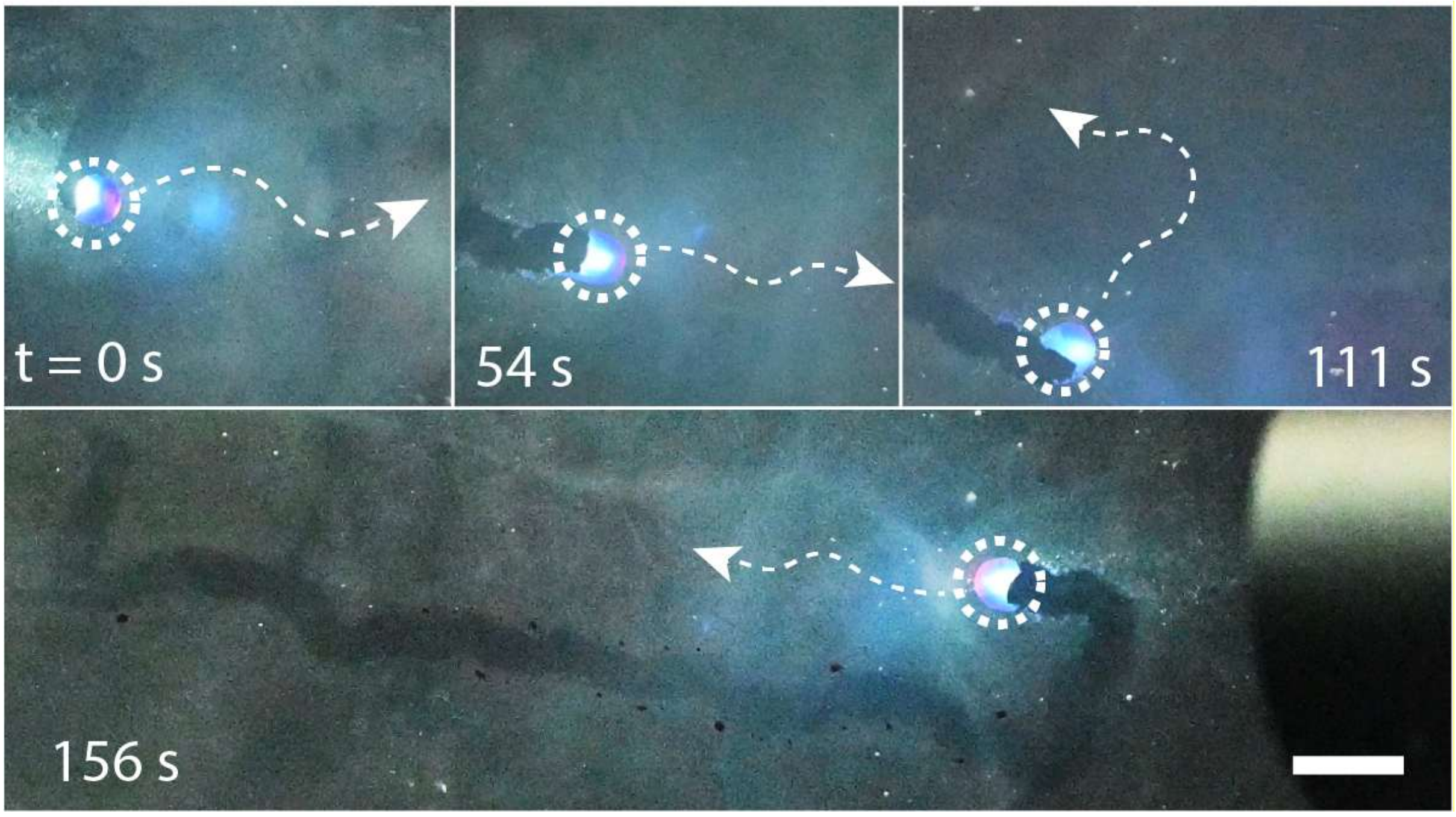

Figure S9. Camera recording of the movement of a UV-responsive droplet (1.0 μL) through a crystalline layer, upon exposure to a handheld UV flashlight (Tank007 UV) with an approx. 1x1 mm^2 beam size. The crystalline layer disintegration occurs mostly at the side of the droplet that is exposed to UV (highlighted as white spot on the droplet), causing the droplet to move away from the UV beam while leaving behind the open channel in the crystalline layer that is visible as a dark trail. The head of the UV flashlight is visible on the right side of the image at t = 156 s. The movie was recorded at 24 fps. The scale bar represents 5 mm.

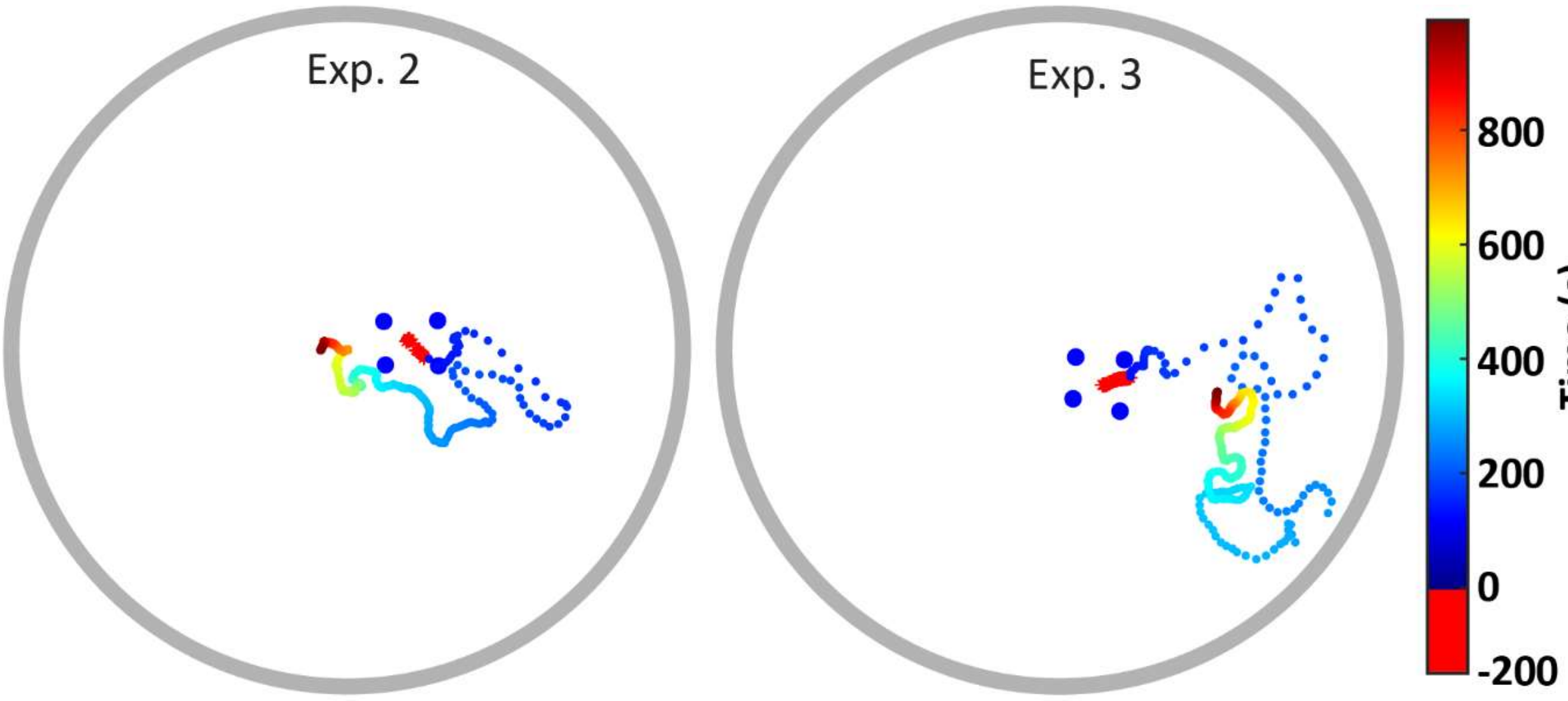

Figure S10. Time-resolved positioning traces corresponding to replicate experiments 2 and 3 of Figure 4b-e. The path of the UV-responsive droplet (1.0 μL) upon UV exposure is depicted in red (t < 0 s); after merging with one of the $C_{12}E_3$ droplets (0.5 μL) at t = 0 s, the trajectory of the merged droplet is depicted in the colour gradient. The grey circles outline the perimeter of the Petri dish (diameter 10 cm).

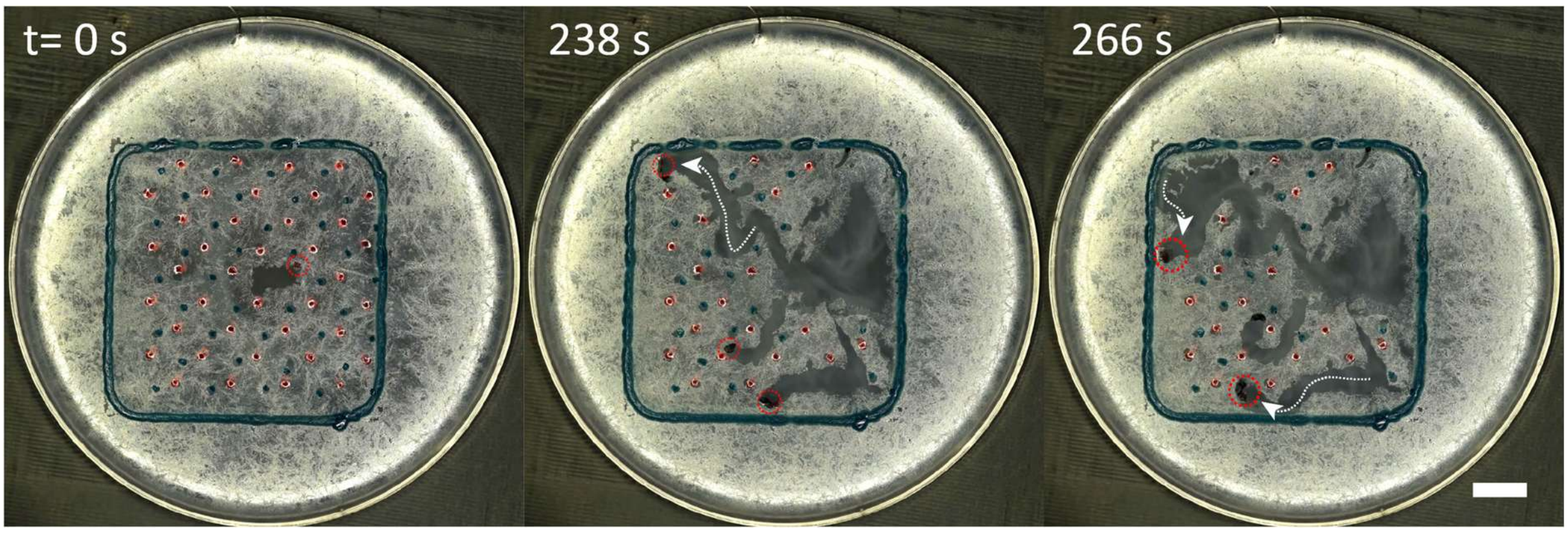

Figure S11. Camera recordings to visualize the crystalline layer around the repellent boundary. A 9x9 droplet grid consisting of UV-responsive droplets (0.4 µL, red) and $C_{12}E_3$ droplets (0.2 µL, blue) is deposited on the crystalline layer in an alternating configuration and confined by a square-shaped repellent boundary ($C_{12}E_3$ in DA, 60% v/v; blue) – in analogy to the experiment shown in Figure 5 of the main text. A circular LED light was used to visualize the crystalline layer (see Methods). Merging a pair of red and blue droplets at t = 0 s produces a motile merged droplet (highlighted with a red dotted circle) that initiates the cascade. The path created by the moving droplet is visualized as dark trail. At t = 238 s, the merged droplet approaches the repellent boundary, where its trajectory is redirected back toward the droplet grid, allowing the process to continue. The scale bar represents 10 mm.

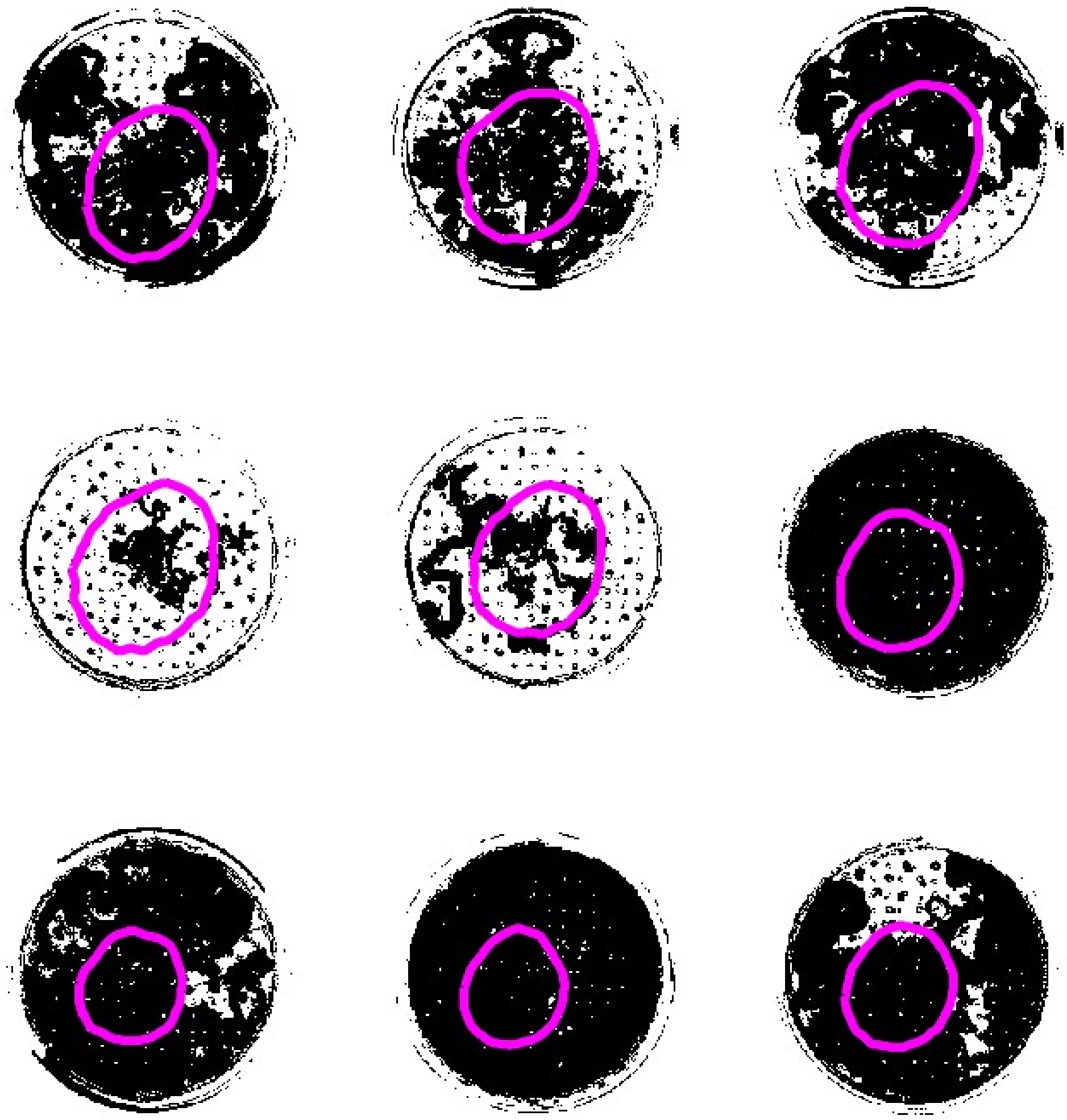

Figure S12. (top) Image analysis of the camera recordings on 9 replicate experiments of a 185-membered droplet grid covering a 10 cm wide Petri dish, consisting of 88 UV-responsive droplets (0.4 µL, red) and 97 $C_{12}E_3$ droplets (0.2 µL, blue) arranged in a 1:1 alternating pattern and confined within a circular repellent boundary deposited on the crystalline layer – corresponding to Movie S5. The black areas represent the pixel locations that have acquired at least once over the time course of the experiment a darker appearance in comparison to the first frame of the respective camera recording (*i.e.* at t = 0 s), assessed via a custom-made MATLAB script. We assume that this can primarily be ascribed to the trajectories of dark-coloured merged droplets travelling across the a/w interface. (We note that scattering effects at the boundary of the Petri dish and the individual red or blue droplets cause minor artefacts in the analysis). The magenta lines indicate the perimeter of the UV-irradiated zone in the first 60 s of the experiments.

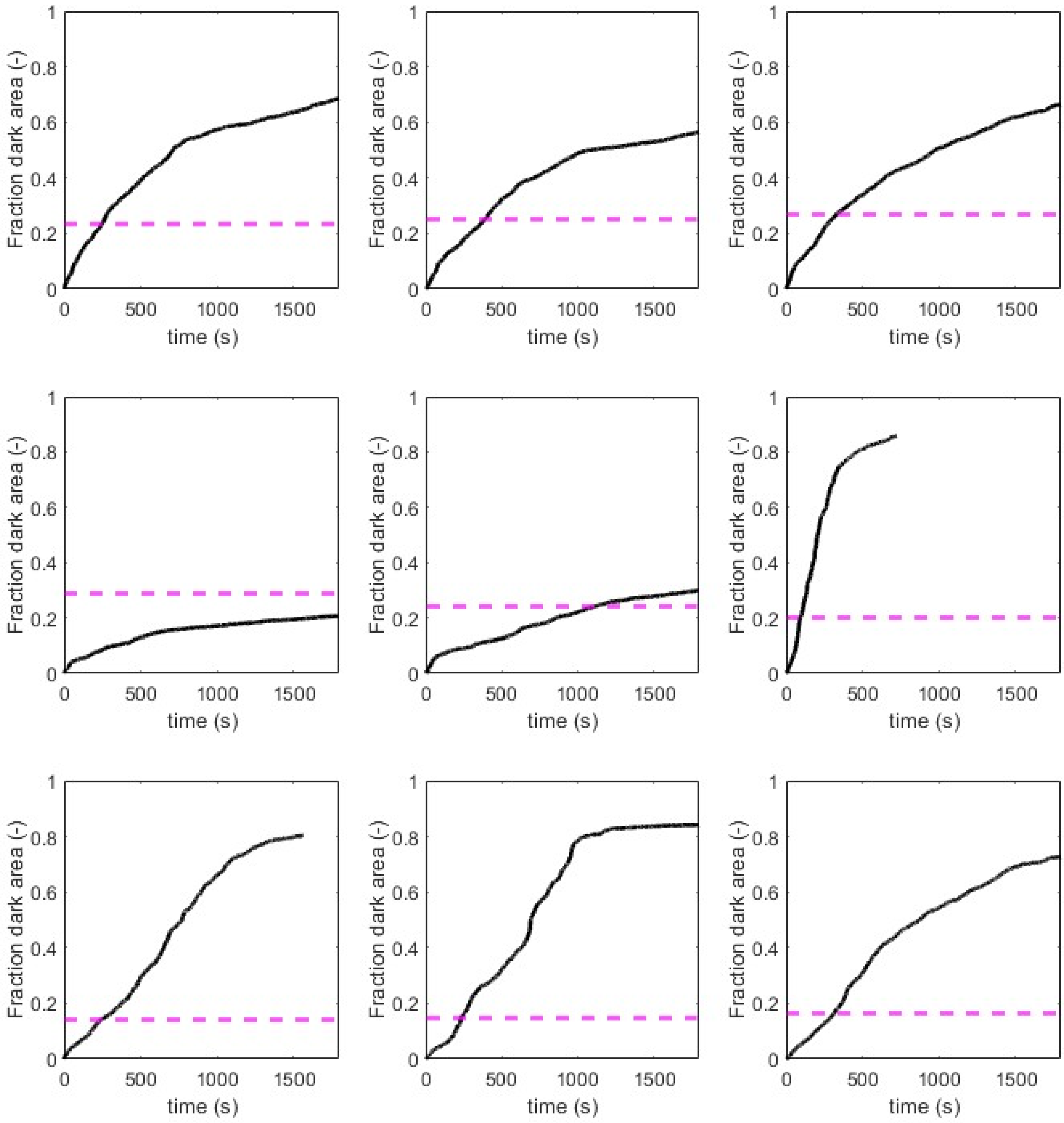

Figure S12 (continued). Graphs of the time-dependent fraction of the black areas (*vide supra*) compared to the total area of the 10 cm wide Petri dish. The dashed magenta lines indicate the area of the UV-irradiated zone in the first 60 s of the experiments.

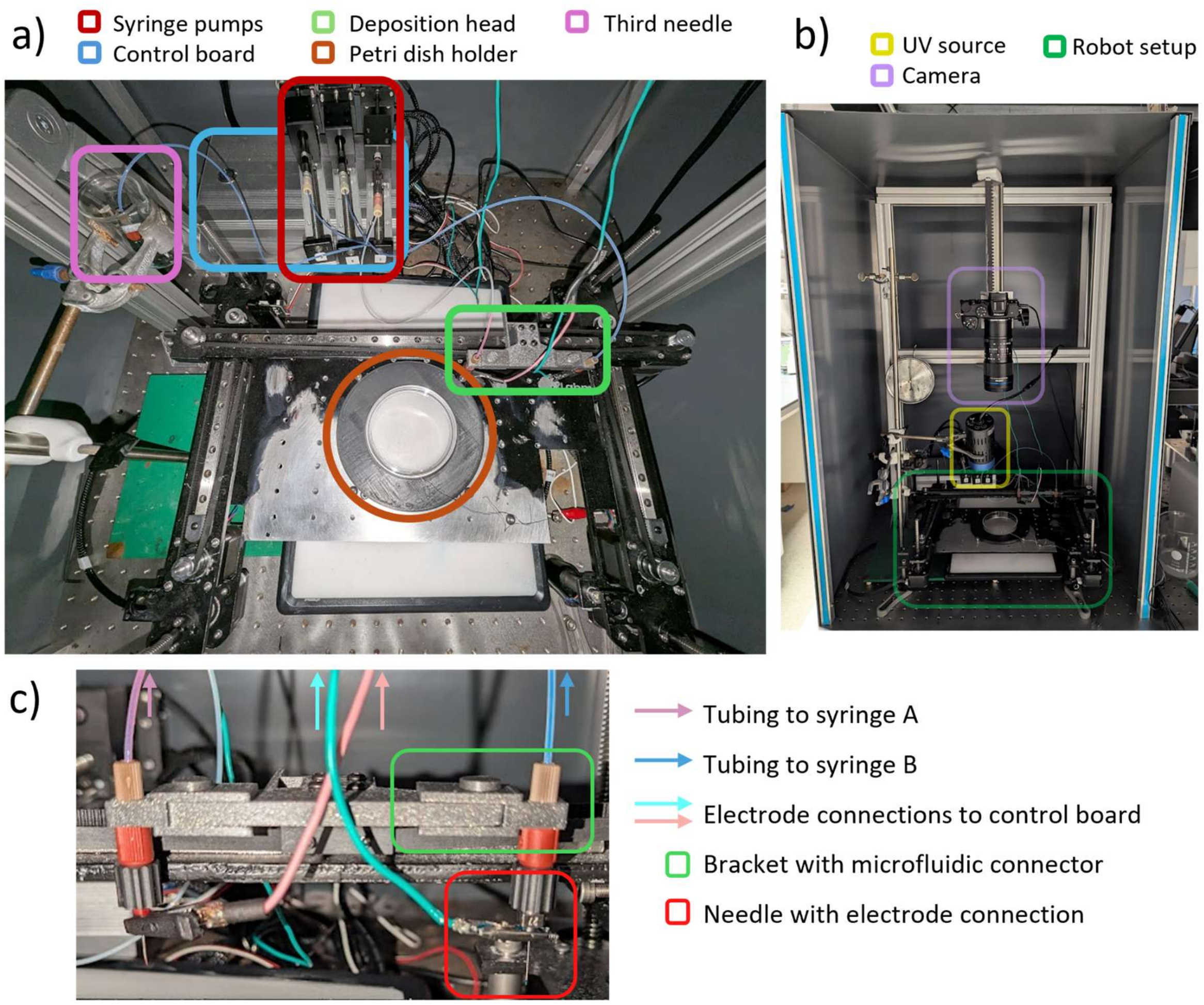

Figure S13. a) Top view of the droplet deposition robot with its components indicated with different colours. b) Front view of the entire setup, including camera, UV source, robot setup and cover box. c) Elements of the deposition head equipped with two needles.

Description of Movies S1 – S5

Movie S1 – Optical microscopy recordings corresponding to Figure 2b and c. (left) Formation of a crystalline layer at the a/w interface of a 3.0 mM DA aqueous solution (6 mL) in a 35 mm Petri dish (Fig. 2b). (right) A UV-responsive droplet (0.5 µL) moves through a crystalline layer under continuous UV exposure of the entire Petri dish (indicated by purple box). The motile droplet consumes the crystalline layer, generating a millimetre-wide channel along its path. Over time, the crystalline layer gradually regrows.

Movie S2 – Camera recording corresponding to Figure 2f. Three replicate experiments of a UV-responsive droplet (0.5 µL) exhibiting run–halt motion on a crystalline layer formed on a large size Petri dish (100 mm) under 30 s on–off UV cycles.

Movie S3 – Optical microscopy and camera recording corresponding to Figure 4. 4 $C_{12}E_3$ droplets (0.5 µL, blue) and a UV-responsive droplet (1.0 µL, red) are deposited on the crystalline layer at the a/w interface, the entire area is exposed to UV until the UV-responsive droplet merges with one of the $C_{12}E_3$ droplets. (top row) Camera recordings on 3 replicate experiments in a large size Petri dish (100 mm). (bottom row) Optical microscopy recording on an experiment conducted in a 35 mm size Petri dish.

Movie S4 – Optical microscopy and camera recordings corresponding to Figure 5. (left) Camera recording on the droplet deposition robot depositing subsequently the repellent boundary (60 v/v % $C_{12}E_3$ in DA, cyan) and the alternating grid of UV-responsive (0.4 µL, red) and $C_{12}E_3$ droplets (0.2 µL, blue) on a crystalline layer formed in a large size Petri dish (100 mm). (right) Optical microscopy recording on the interaction of manually deposited $C_{12}E_3$ droplet (0.5 µL), mesitylene droplet with 5% DA (1.0 µL) and repellent boundary. (No PAG was included in the red droplet, the mesitylene and $C_{12}E_3$ droplets were intentionally merged using a pipette tip).

Movie S5 – Camera recordings corresponding to Figure 6b-c. Camera recordings on 9 replicate experiments of a droplet grid covering a large size Petri dish (100 mm), consisting of 88 UV-responsive droplets (0.4 µL, red) and 97 $C_{12}E_3$ droplets (0.2 µL, blue) arranged in a 1:1 alternating pattern and confined within a circular repellent boundary deposited on the crystalline layer. A subset of the droplets is exposed to UV for approx. 60 seconds, the resulting merged droplets initiate a cascade of motile droplets.
